# Molecular mechanism of BK channel activation by the smooth muscle relaxant NS11021

**DOI:** 10.1085/jgp.201912506

**Published:** 2020-03-27

**Authors:** Michael E. Rockman, Alexandre G. Vouga, Brad S. Rothberg

**Affiliations:** Department of Medical Genetics and Molecular Biochemistry, Temple University Lewis Katz School of Medicine, Philadelphia, PA

## Abstract

Large-conductance Ca^2+^-activated K^+^ channels (BK channels) are activated by cytosolic calcium and depolarized membrane potential under physiological conditions. Thus, these channels control electrical excitability in neurons and smooth muscle by gating K^+^ efflux and hyperpolarizing the membrane in response to Ca^2+^ signaling. Altered BK channel function has been linked to epilepsy, dyskinesia, and other neurological deficits in humans, making these channels a key target for drug therapies. To gain insight into mechanisms underlying pharmacological modulation of BK channel gating, here we studied mechanisms underlying activation of BK channels by the biarylthiourea derivative, NS11021, which acts as a smooth muscle relaxant. We observe that increasing NS11021 shifts the half-maximal activation voltage for BK channels toward more hyperpolarized voltages, in both the presence and nominal absence of Ca^2+^, suggesting that NS11021 facilitates BK channel activation primarily by a mechanism that is distinct from Ca^2+^ activation. 30 µM NS11021 slows the time course of BK channel deactivation at −200 mV by ∼10-fold compared with 0 µM NS11021, while having little effect on the time course of activation. This action is most pronounced at negative voltages, at which the BK channel voltage sensors are at rest. Single-channel kinetic analysis further shows that 30 µM NS11021 increases open probability by 62-fold and increases mean open time from 0.15 to 0.52 ms in the nominal absence of Ca^2+^ at voltages less than −60 mV, conditions in which BK voltage sensors are largely in the resting state. We could therefore account for the major activating effects of NS11021 by a scheme in which the drug primarily shifts the pore-gate equilibrium toward the open state.

## Teaser

Large-conductance Ca^2+^-activated K^+^ channels (BK channels) are key drug targets due to their association with a wide variety of neurological disorders. Rockman et al. reveal that the smooth muscle relaxant NS11021 activates BK channels by shifting the pore-gate equilibrium toward the open state.

## Introduction

Large-conductance Ca^2+^-activated K^+^ channels (BK channels) are expressed in a wide variety of tissues, including neurons, vascular smooth muscle, and secretory epithelium, where they have been implicated in both control of neuronal firing rates and action potential duration, regulation of smooth muscle contractility and vascular tone, and control of the resting potential, respectively (Latorre et al., 2017). The role of BK channels in control of vascular tone is well established; thus, targeting of BK channels by pharmacological activators may have the potential to treat conditions such as ischemia-reperfusion injury, overactive bladder, and erectile dysfunction. In addition, BK activators may be useful as therapeutics in disease where decreased BK channel function is an underlying factor, and these activators may also serve as research tools to understand and probe molecular mechanisms of channel gating (Bentzen et al., 2014; Kshatri et al., 2018; Koval et al., 2007; Hoshi and Heinemann, 2016).

Structurally, BK channels are tetramers in which each subunit consists of an N-terminal voltage-sensing domain (VSD; transmembrane helices S0–S4), a pore/gate domain (PGD; transmembrane helices S5-P-S6), and a Ca^2+^-sensing domain (CSD) comprised of two tandem cytosolic RCK domains (Giraldez and Rothberg, 2017). Recently, it was suggested that the BK channel opener Cym04, a dehydroabietic acid derivative, as well as the benzimidazolone derivative NS1619, may act through a mechanism that involves binding to the S6-RCK linker (Gessner et al., 2012). A neuronal BK splice variant (slo1_9a) that contains alternative splicing in the S6-RCK linker is insensitive to Cym04, and activation by Cym04 can be abrogated by a two-residue deletion in this linker. In addition to NS1619 and Cym04, several other compounds comprising a diverse range of chemical classes are known BK channel activators, including the natural products mallotoxin and dehydroxysoyasaponin, and synthetic compounds such as the aryloxindole derivative BMS-204352 and the biarylthiourea derivative NS11021. Compounds comprising such a wide array of structures may contain distinct pharmacophores and may thus have distinct structural or functional actions that may be exploited for therapeutic purposes. It will be important to identify molecular mechanisms for each of these diverse compounds to improve understanding of BK channel gating mechanisms (Horrigan et al., 1999; Horrigan and Aldrich, 2002; Rothberg et al., 1997; Rothberg and Magleby, 1999; Cox et al., 1997; Cui and Aldrich, 2000).

Here we have focused on the mechanism of NS11021, a potent and relatively specific BK activator that has been studied in animal models for treatment of ischemia-reperfusion injury and erectile dysfunction through its vasodilatory effects (Kun et al., 2009; Bentzen et al., 2007, 2009). Using patch-clamp electrophysiology, we found that NS11021 shifts the voltage dependence of activation primarily through slowing of the overall channel closing rate. This action of NS11021 persists in the nominal absence of Ca^2+^ in hslo1-WT channels, as well as in truncated BK channels from which the CSD has been deleted (slo1c-Kv-minT). Addition of NS11021 leads to an increase in BK channel open probability (P_o_) at negative voltages under conditions where the VSD is largely in the resting state, consistent with the idea that NS11021 is acting at the PGD. This action is distinct from the effects of either of the structurally different BK activators Cym04 or NS1619 (Gessner et al., 2012). We can account for the major features of activity of NS11021 through a kinetic scheme in which the drug acts by slowing transition rates of the PGD toward the closed state by approximately fivefold.

## Materials and methods

### Electrophysiology

Experiments were performed using excised inside-out patches from human embryonic kidney cells (HEK-293T; American Type Culture Collection) stably transfected with human BK channel α-subunit cDNA (referred to as HF1 cells; Meera et al., 1997), obtained from the laboratory of Dr. R. Aldrich (University of Texas, Austin, TX). Slo1c-Kv-minT (in pcDNA3.3; gift of Dr. T. Giraldez, University of La Laguna, Santa Cruz de Tenerife, Spain; Budelli et al., 2013; Webb et al., 2015), was overexpressed in HEK-293T cells following transient transfection by electroporation.

Except where noted, solutions bathing both sides of the membrane contained 160 mM KCl and 10 mM HEPES (pH 7.4). For data in the presence of 1 or 10 µM free Ca^2+^, solutions at the cytoplasmic face of the patch additionally contained 2 mM *N*-(2-hydroxyethyl)-ethylene-diamine-triacetic acid, with 0.394 or 1.429 mM CaCl_2_ added to bring the free [Ca^2+^] to the indicated levels, respectively, for pH 7.4, *T* = 22°C, and ionic strength = 0.16 (Bers et al., 2010). For patches with 100 µM free Ca^2+^, the solution contained 2 mM nitrilotriacetic acid as a calcium buffer, with 0.972 mM CaCl_2_. For experiments at nominally 0 free Ca^2+^, the solution contained 5 mM EGTA with no added CaCl_2_ (free [Ca^2+^] estimated to be <0.3 nM; Bers et al., 2010).

NS11021 was included in solutions applied to the cytoplasmic face of the patch. NS11021 powder (Tocris Bioscience) was dissolved in DMSO to make a 100-mM stock solution, which was then aliquoted and further dissolved to yield a series of less concentrated stock solutions, so that the final concentration of DMSO in all experiments was 0.1% (vol/vol). All experiments performed in the absence of NS11021 also included 0.1% DMSO (vol/vol, final concentration), which was observed to have no effect on BK channel gating compared with experiments performed in DMSO-free solutions.

Patch-clamp experiments were performed at room temperature (22°C), using a Dagan PC-ONE amplifier controlled by pClamp9 software. Solutions at the cytosolic face of the excised inside-out membrane patch were typically changed multiple times during the course of each experiment in a gravity-fed perfusable recording chamber, to measure BK channel activity over a range of [Ca^2+^] and [NS11021]. To minimize voltage errors arising from series resistance, we analyzed only recordings in which the maximal current was <4 nA. We estimate that the maximal voltage error contributed by series resistance for these recordings was 6 mV, and analyzed data are presented without correction for series resistance.

### Data analysis

Following patch excision, channel activity was assessed by acquisition of currents in response to a series of voltage steps, from a holding voltage of typically −90 mV to test voltages ranging from −80 to 250 mV (100 ms) to yield a range of activity levels, and then back to typically −60 mV to elicit a tail current. Each voltage protocol was typically repeated at least five times for each patch, and current traces were digitized and averaged in real time using pClamp9 software. Tail-current amplitudes were measured, plotted as a function of test voltage, and fitted with a Boltzmann equation:

$$G/G_{\max}=\frac{1}{1+\exp\left[\frac{-z\left(V-V_{1/2}\right)}{k_{B}T}\right]},$$ (1)

where *G*/*G*_max_ is the normalized tail current amplitude, *z* is the effective gating valence, *V*_1/2_ is the voltage at half-maximal activation, *k_B_* is Boltzmann’s constant, and *T* is temperature.

**Figure S1.**
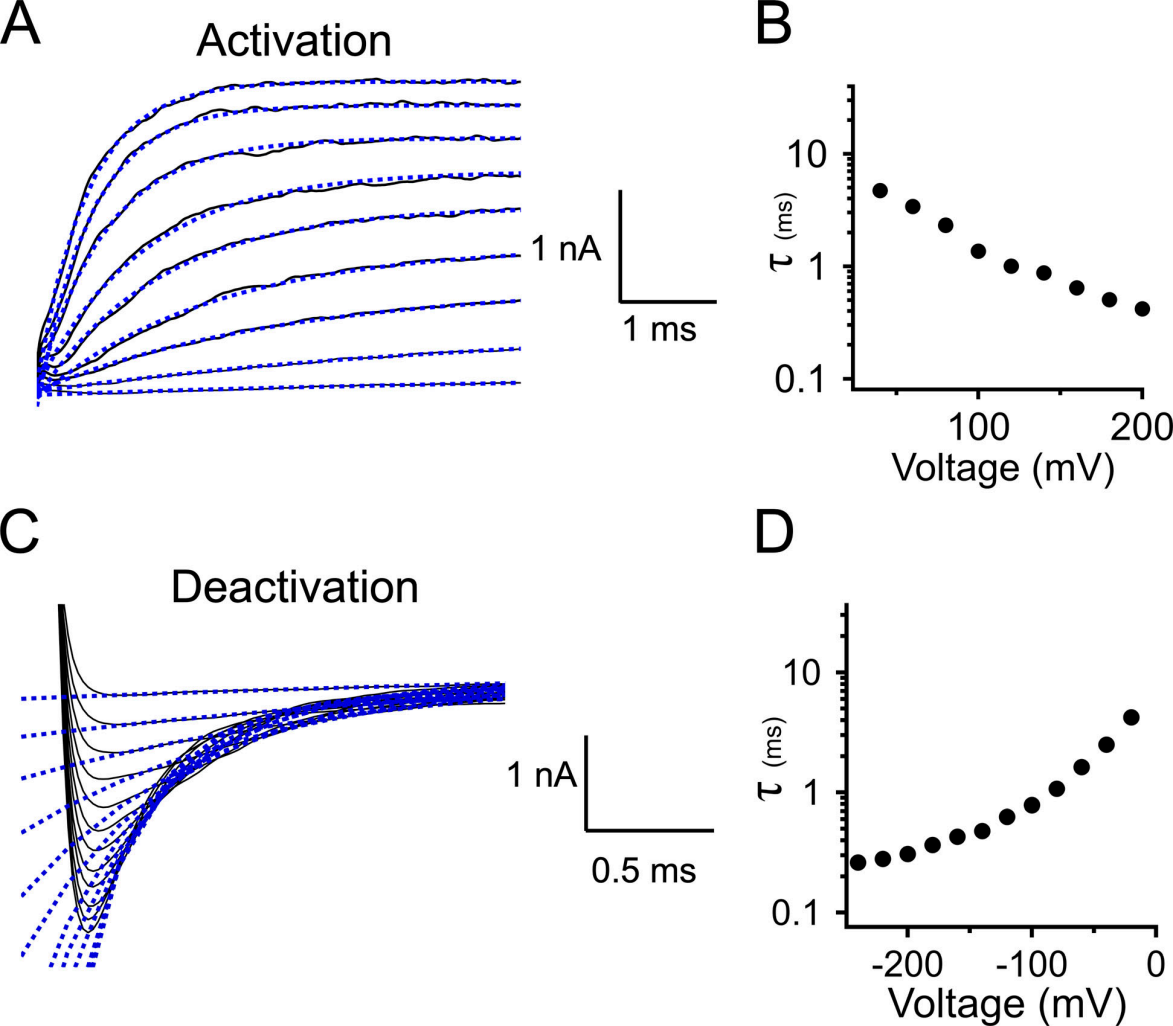
**Representative fits of BK channel activation and deactivation time course with a single exponential for currents with 10 µM Ca^2+^ at the cytosolic side of the patch.** **(A)** Representative macroscopic currents (black lines), activated by voltage step from −100 mV to between +40 mV and +200 mV (20-mV increments). Single exponential fits (Eq. 2) are shown as blue dashed lines. **(B)** Time constants corresponding to the fits in A, plotted as a function of voltage. **(C)** Representative macroscopic currents (black lines), driven by voltage steps from +100 mV to between −20 mV and −240 mV (20-mV increments). Single exponential fits are shown as blue dashed lines. **(D)** Time constants corresponding to the fits in C plotted as a function of voltage.

The time constant of current relaxation (τ) was quantified over a range of voltages for both channel activation and deactivation using standard voltage-step protocols (Horrigan et al., 1999). For activation kinetics, patches were initially held at a hyperpolarized voltage at which channels are mainly closed, before stepping to a series of depolarizing voltages; for deactivation kinetics, patches were conversely held at depolarized voltage to maximally activate the channels, followed by steps to a series of hyperpolarizing voltages. Currents were fitted directly with a single exponential function,

$$I=Aexp\left(\frac{-t}{\tau }\right)+y_{0},$$ (2)

where *I* is the current, *A* is the amplitude, *t* is time, τ is the time constant of relaxation, and *y*_0_ is the level of steady-state current. We have found that under the conditions of our experiments, BK current relaxations were described well by a single exponential over a wide range of voltages, as observed previously (Fig. S1; Horrigan et al., 1999; Cui et al., 1997).

For single-channel recordings, dwell times were measured using 50% threshold detection and log-binned at 10 bins per log unit using pClamp9 (Sigworth and Sine, 1987; McManus et al., 1987). Dwell-time histograms were fitted with sums (mixtures) of exponential components from a lower limit of twice the system dead time (0.036 ms) using the maximum likelihood method (Sigworth and Sine, 1987; Horn and Lange, 1983; McManus et al., 1987). The number of active channels (N) per patch was estimated by dividing the maximum macroscopic tail current amplitude for the patch by the single channel current amplitude at the same voltage (Koval et al., 2007; Wang et al., 2006, 2009). The P_o_ per channel was calculated by dividing the measured NP_o_ by N.

The relation between [NS11021] and channel activity was analyzed by plotting *G*/*G*_max_ as a function of [NS11021] for a given voltage and fitting with a Hill equation,

$$G/G_{\max}=min+\frac{1\;-\;min}{1+{\left(\frac{{\mathrm{EC}}_{50}}{\left[NS11021\right]}\right)}^{n_{H}}},$$ (3)

where *min* is the minimum *G*/*G*_max_ at that voltage, EC_50_ is the concentration at half-maximal response, and *n_H_* is the Hill coefficient. To obtain reasonably well-constrained parameter estimates, this analysis was limited to datasets (voltages) in which the minimum *G*/*G*_max_ in the absence of NS11021 was <0.5, and the highest *G*/*G*_max_ in the presence of NS11021 was >0.5.

Data across different patches are presented as means ± SEM. The experimental data presented represent results from a total of 160 different patches.

### Kinetic modeling

To analyze the mechanism underlying effects of NS11021 on the gating of BK channels, conductance–voltage (G–V) curves in the presence of various concentrations of Ca^2+^ were fitted with a dual allosteric model (Horrigan and Aldrich, 2002):

$$P_{o}=\;\frac{1}{1+\;\frac{{\left(1+J+K+JKE\right)}^{4}}{L{\left(1+KC+JD+JKCDE\right)}^{4}}},$$ (4)

where *J* = *J*_0_exp(*z_J_V*/*k_B_T*), *L* = *L*_0_exp(-*z_L_V*/*k_B_T*), and *K* = [Ca^2+^]/*K_D_*. For these equations, *J*_0_ represents the equilibrium constant for voltage sensor movement at 0 mV with *z_J_* as the effective valence, *L*_0_ is the closed-to-open pore equilibrium constant with *z_L_* as the effective valence, and *K_D_* represents the dissociation constant for Ca^2+^. The allosteric coupling constants *C*, *D*, and *E* represent the interaction between Ca^2+^ binding and opening, voltage sensor activation and opening, and Ca^2+^ binding and voltage sensor movement, respectively, as illustrated in Scheme 1 (Horrigan et al., 1999; Horrigan and Aldrich, 2002; Ma et al., 2006; Wang et al., 2006; Koval et al., 2007).

**(Scheme 1):**
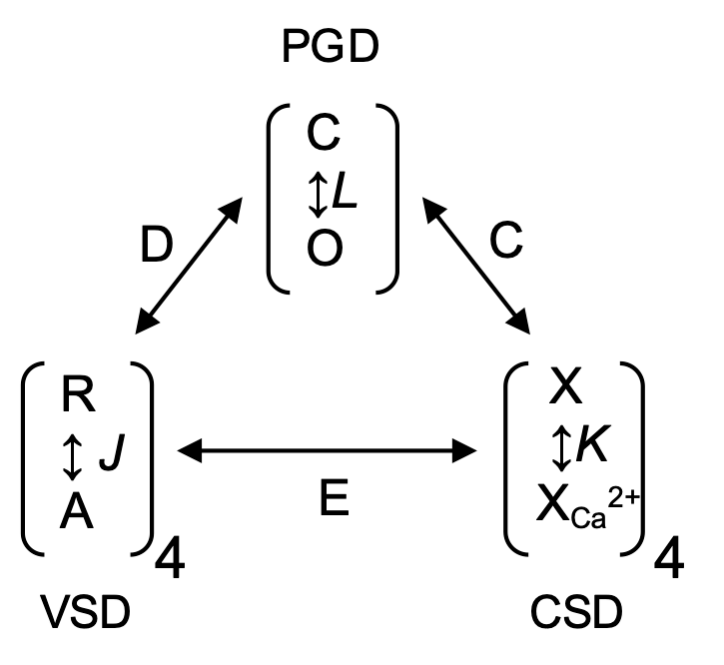


For our analysis, sets of values for the model parameters in Eq. 4 were first estimated using a range of different initial starting parameters, to minimize the value of χ^2^ determined from comparison with mean G–V curves and P_o_ data at nominally 0, 1, 10, and 100 µM Ca^2+^, all at 0 µM NS11021. This was achieved by global fitting using the Levenberg–Marquardt algorithm, implemented in the Global Fit package in IgorPro 8 (WaveMetrics), in which

$$\chi^{2}=\overset{n}{\underset{i=1}{\sum }}{\left(\frac{Y_{i}-{\hat{Y}}_{i}}{\sigma_{i}}\right)}^{2},$$

where *Y_i_* and ${\hat{Y}}_{i}$ are the *i*th observed and corresponding predicted values, and σ*_i_* is the observed variance. Based on this approach, two “standard” parameter sets (Fit A and Fit B) were selected, each yielding very similar values of χ^2^ and thus statistically equivalent descriptions of the BK channel G–V data with 0 µM NS11021. These two parameter sets were used in fitting datasets in the presence of 0.1, 1, 10, and 30 µM NS11021 by the following approach. A dataset at a given [NS11021] (at nominally 0, 1, 10, and 100 µM Ca^2+^) was fitted, again using the Global Fit package in IgorPro 8, by allowing only one parameter to be adjusted (i.e., only *J*_0_, *K_D_*, *L*_0_, *z_L_*, *D*, etc.). Thus, estimates of each single parameter were obtained as a function of [NS11021] against the set of standard Fit A or Fit B parameters.

The mechanism of NS11021 action on voltage-dependent gating at nominally 0 Ca^2+^ was further analyzed using a 10-state voltage-dependent scheme Scheme 2, which used microscopic rate constants rather than equilibrium constants, to enable calculation of activation and deactivation time constants for BK channel gating. This is similar to approaches used in previous work (Horrigan et al., 1999), except that Scheme 2 was based on the simplifying constraint that all closed-open (C-O) transitions were allosterically coupled to independently activated VSDs.

**(Scheme 2):**
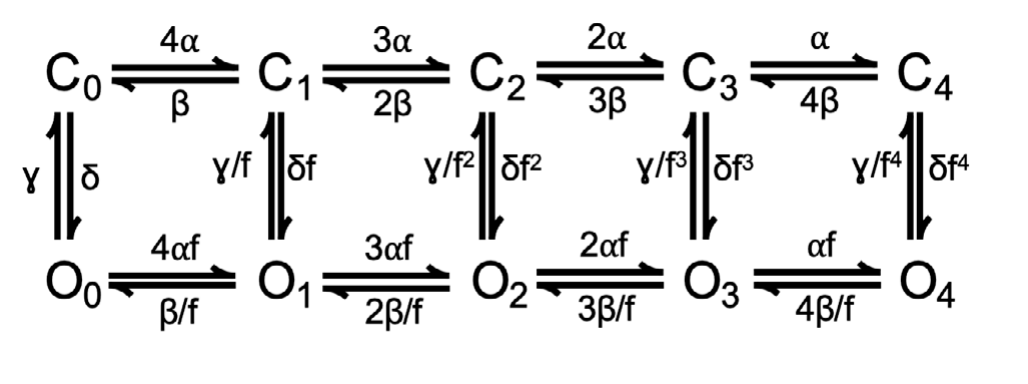


In Scheme 2, rate constants were defined by rates for VSD activation and deactivation (α and β, respectively), PGD closing and opening (γ and δ), a gating valence for each of these rate constants (*z*_α_, *z*_β_, *z*_γ_, and *z*_δ_), and allosteric coupling between the VSD and PGD (*D*) using the additional simplifying assumption *D* = *f*^2^ (i.e., VSD-activation coupling to PGD opening is energetically equivalent to VSD-deactivation coupling to PGD closing). Effective rates were calculated at each voltage as

$$rate_{ij}\left(V\right)\;=\;rate_{ij}\left(0\right)\exp\left(z_{ij}V/k_{B}T\right),$$ (5)

in which *rate_ij_* denotes the rate from state *i* to state *j*. For example, if α = 100 and *z*_α_ = 1.5, then the effective rate from *C*_0_ to *C*_1_ in Scheme 2 = 4[100exp(1.5*V*/*kT*)] (Weiss and Magleby, 1992; Zagotta et al., 1994; Rothberg and Magleby, 2000). Using Scheme 2, the principal (i.e., rate-limiting) time constants for voltage-dependent activation were calculated over a range of voltages using Q-matrix methods (Colquhoun and Hawkes, 1995) in IgorPro 8. These were compared with experimentally determined time constants by χ^2^ as defined above, and model parameters were optimized through subsequent iterations using a direct search approach based on Patternsearch (Colquhoun, 1971).

### Online supplemental material

**Figure S2.**
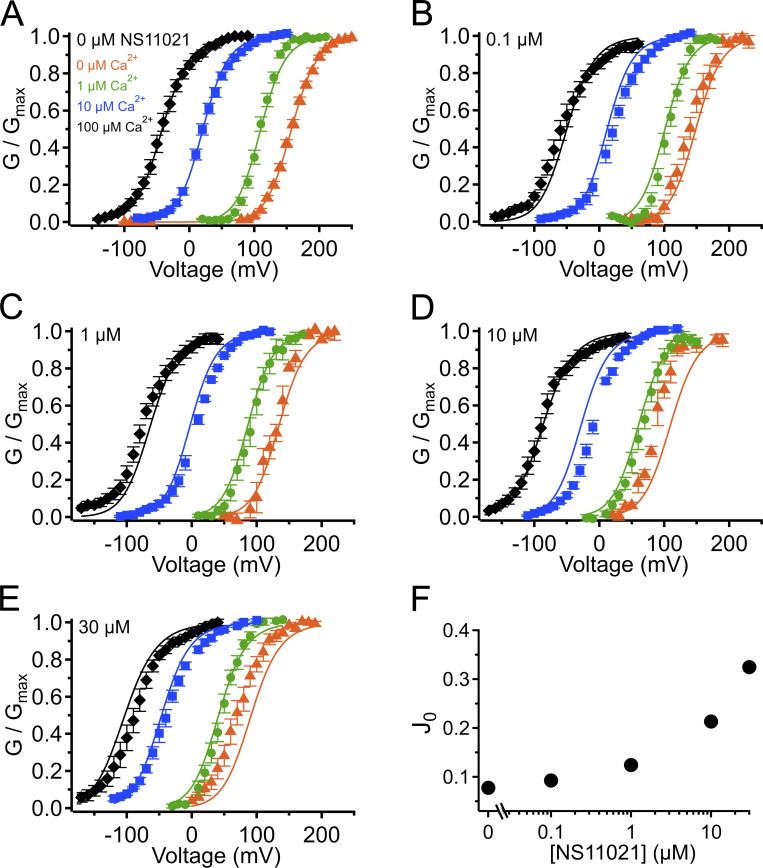
**G–V-Ca^2+^ relations with predictions from** **Scheme 1** **using parameters from Fit A, with adjustable parameter *J*_0_.** **(A)** G–V-Ca^2+^ relations in the absence of NS11021 bathed in 0 µM (orange triangle), 1 µM (green circle), 10 µM (blue square), and 100 µM Ca^2+^ (black diamond). Parameters are listed under Fit A in Table 1. Lines represent the predictions with *J*_0_ = 0.08. **(B–E)** G–V-Ca^2+^ relations in 0.1, 1, 10, and 30 µM NS11021, respectively. Lines represent predictions with *J*_0_ = 0.09 (0.1 µM), 0.12 (1 µM), 0.21 (10 µM), and 0.32 (30 µM). **(F)** Fitted values for *J*_0_ versus [NS11021]. These suggest that the major effects of NS11021 on G–V-Ca^2+^ relations may be explained in part by an increase in *J*_0_ with increasing [NS11021] (but see more detailed explanation in Results).

**Figure S3.**
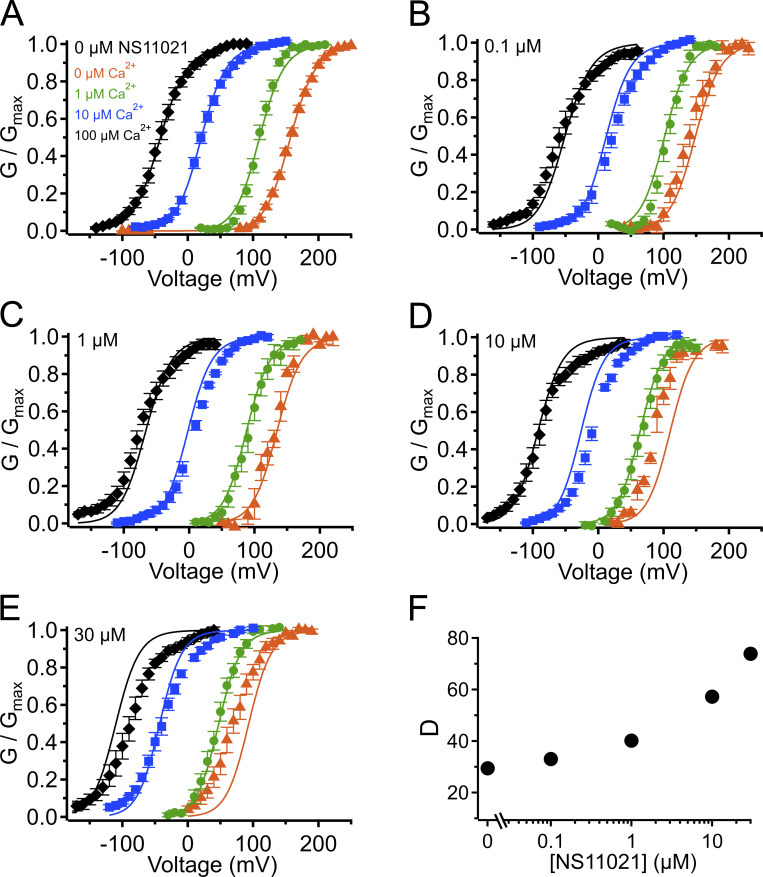
**G–V-Ca^2+^ relations with predictions from** **Scheme 1** **using parameters from Fit A, with adjustable parameter *D*.** **(A)** G–V-Ca^2+^ relations in the absence of NS11021 bathed in 0 µM (orange triangle), 1 µM (green circle), 10 µM (blue square), and 100 µM Ca^2+^ (black diamond). Parameters are listed under Fit A in Table 1. Lines represent the predictions with *D* = 29. **(B–E)** G–V-Ca^2+^ relations in 0.1, 1, 10, and 30 µM NS11021, respectively. Lines represent predictions with *D* = 33 (0.1 µM), 40 (1 µM), 57 (10 µM), and 74 (30 µM). **(F)** Fitted values for *D* versus [NS11021]. These suggest that the major effects of NS11021 on G–V-Ca^2+^ relations may be explained in part by an increase in *D* with increasing [NS11021] (but see more detailed explanation in Results).

**Figure S4.**
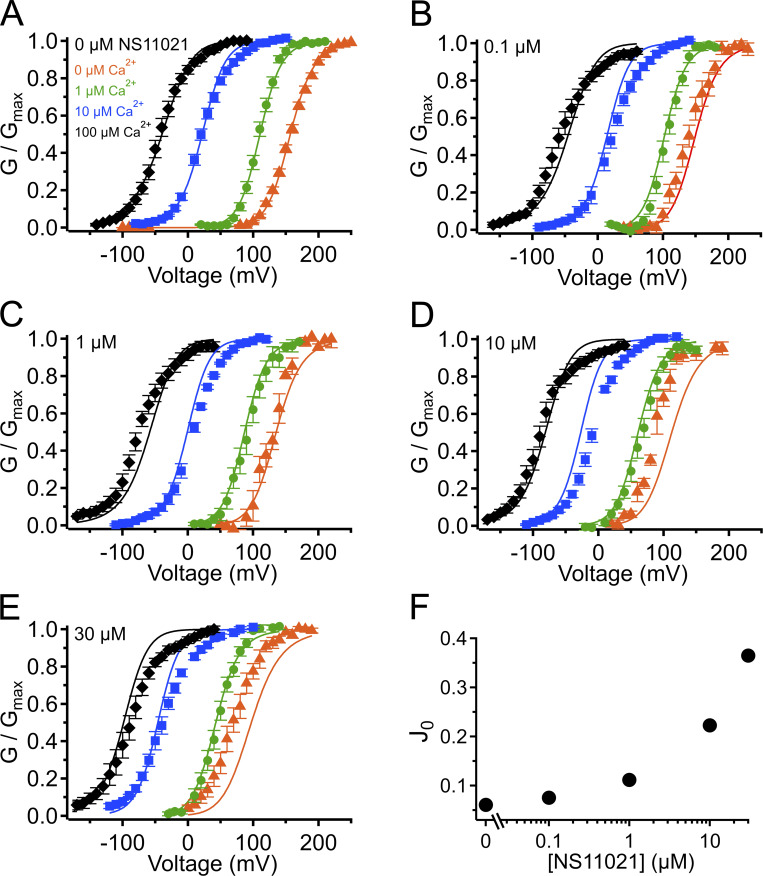
**G–V-Ca^2+^ relations with predictions from** **Scheme 1** **using parameters from Fit B, with adjustable parameter *J*_0_.** **(A)** G–V-Ca^2+^ relations in the absence of NS11021 bathed in 0 µM (orange triangle), 1 µM (green circle), 10 µM (blue square), and 100 µM Ca^2+^ (black diamond). Parameters are listed under Fit B in Table 1. Lines represent the predictions with *J*_0_ = 0.06. **(B–E)** G–V-Ca^2+^ relations in 0.1, 1, 10, and 30 µM NS11021, respectively. Lines represent predictions with *J*_0_ = 0.08 (0.1 µM), 0.11 (1 µM), 0.22 (10 µM), and 0.36 (30 µM). **(F)** Fitted values for *J*_0_ versus [NS11021]. These suggest that the major effects of NS11021 on G–V-Ca^2+^ relations may be explained in part by an increase in *J*_0_ with increasing [NS11021] (but see more detailed explanation in Results).

**Figure S5.**
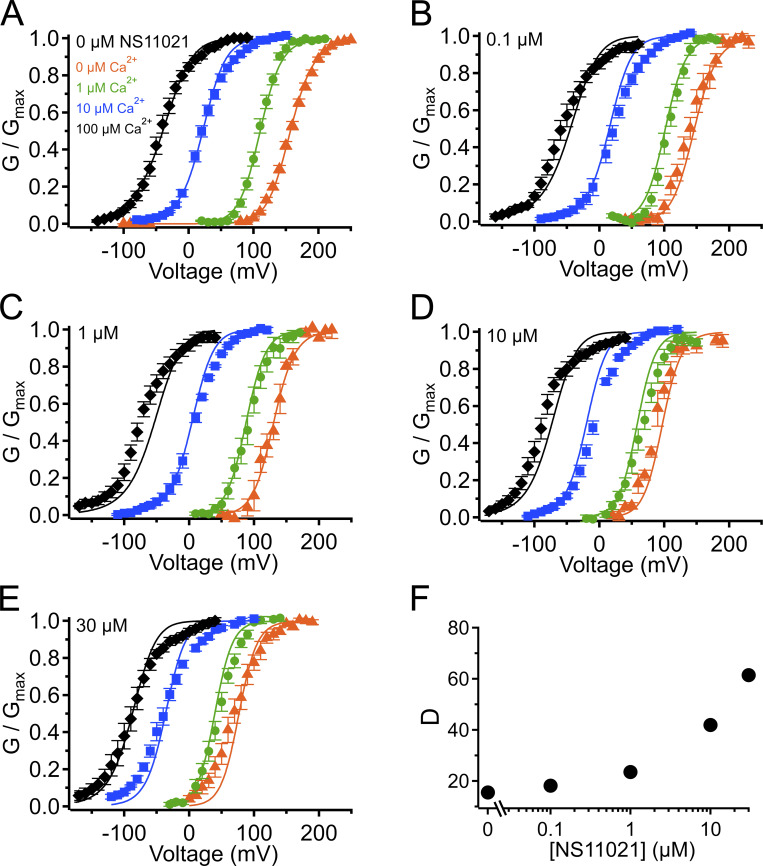
**G–V-Ca^2+^ relations with predictions from** **Scheme 1** **using parameters from Fit B, with adjustable parameter *D*.** **(A)** G–V-Ca^2+^ relations in the absence of NS11021 bathed in 0 µM (orange triangle), 1 µM (green circle), 10 µM (blue square), and 100 µM Ca^2+^ (black diamond). Parameters are listed under Fit B in Table 1. Lines represent the predictions with *D* = 16. **(B–E)** G–V-Ca^2+^ relations in 0.1, 1, 10, and 30 µM NS11021, respectively. Lines represent predictions with *D* = 18 (0.1 µM), 23 (1 µM), 42 (10 µM), and 61 (30 µM). **(F)** Fitted values for *D* versus [NS11021]. These suggest that the major effects of NS11021 on G–V-Ca^2+^ relations may be explained in part by an increase in *D* with increasing [NS11021] (but see more detailed explanation in Results).

**Figure S6.**
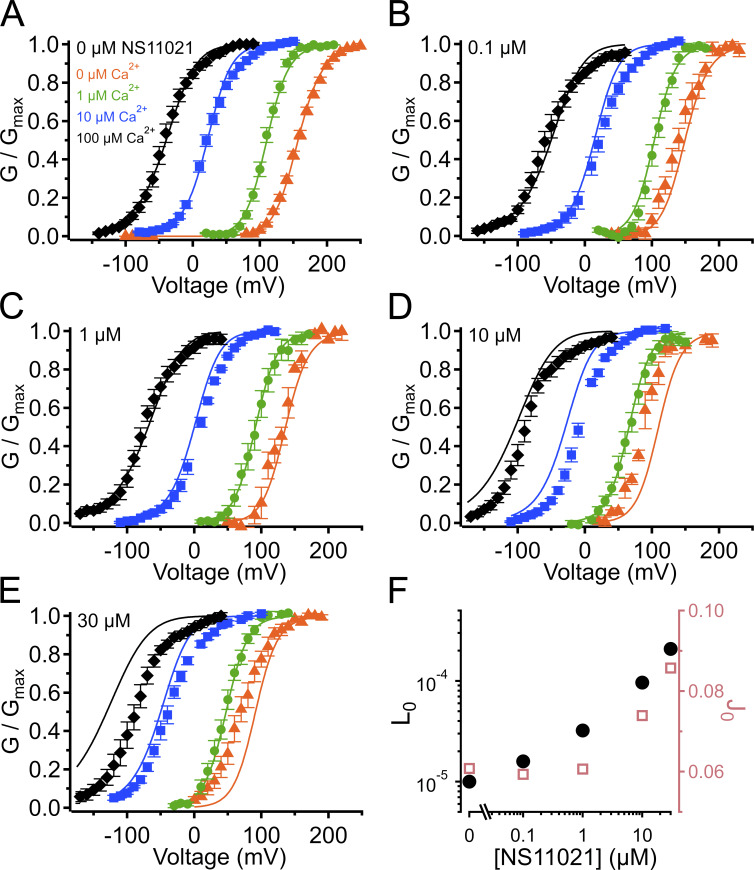
**G–V-Ca^2+^ relations from Scheme 1** **using parameters from Fit B to describe changing parameter *J*_0_ in addition to *L*_0_.** **(A)** G–V-Ca^2+^ relations in the absence of NS11021 bathed in 0 µM (orange triangle), 1 µM (green circle), 10 µM (blue square), and 100 µM Ca^2+^ (black diamond). Parameters are listed under Fit B in Table 1. Lines represent the predictions with *L*_0_ = 1.0 × 10^−5^, *J*_0_ = 0.06. **(B–E)** G–V-Ca^2+^ relations in 0.1, 1, 10, and 30 µM NS11021, respectively. Lines represent predictions with *L*_0_ = 1.6 × 10^−5^, *J*_0_ = 0.06 for 0.1 µM NS11021; *L*_0_ = 3.2 × 10^−5^, *J*_0_ = 0.06 for 1 µM NS11021; *L*_0_ = 9.6 × 10^−5^, *J*_0_ = 0.07 for 10 µM NS11021; *L*_0_ = 2.1 × 10^−4^, *J*_0_ = 0.09 for 30 µM NS11021. **(F)** Fitted values for *L*_0_ (black) and *J*_0_ (red) versus [NS11021]. These suggest that the major effects of NS11021 on G–V-Ca^2+^ relations may be explained in part by an increase in *L*_0_ in combination with an increase in *J*_0_, with increasing [NS11021].

**Figure S7.**
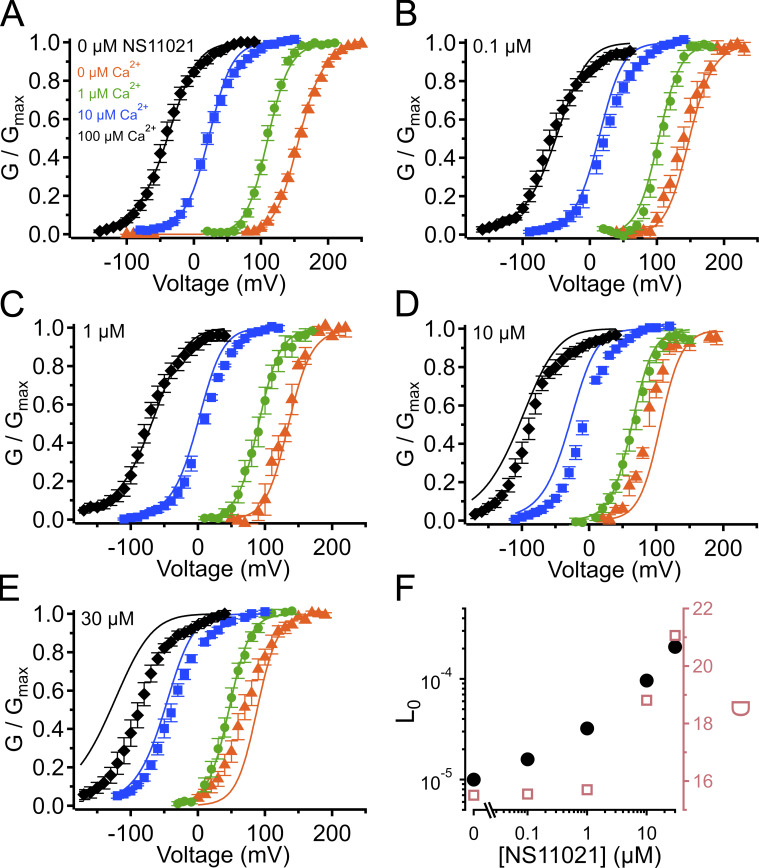
**G–V-Ca^2+^ relations from** **Scheme 1** **using parameters from Fit B to describe changing parameter *D* in addition to *L*_0_.** **(A)** G–V-Ca^2+^ relations in the absence of NS11021 bathed in 0 µM (orange triangle), 1 µM (green circle), 10 µM (blue square), and 100 µM Ca^2+^ (black diamond). Parameters are listed under Fit B in Table 1. Lines represent the predictions with *L*_0_ = 1.0 × 10^−5^, *D* = 15. **(B–E)** G–V-Ca^2+^ relations in 0.1, 1, 10, and 30 µM NS11021, respectively. Lines represent predictions with *L*_0_ = 1.6 × 10^−5^, *D* = 16 for 0.1 µM NS11021; *L*_0_ = 3.2 × 10^−5^, *D* = 16 for 1 µM NS11021; *L*_0_ = 9.6 × 10^−5^, *D* = 19 for 10 µM NS11021; *L*_0_ = 2.1 × 10^−4^, *D* = 21 for 30 µM NS11021. **(F)** Fitted values for *L*_0_ (black) and *D* (red) versus [NS11021]. These suggest that the major effects of NS11021 on G–V-Ca^2+^ relations may be explained in part by an increase in *L*_0_ in combination with an increase in *D*, with increasing [NS11021].

**Figure S8.**
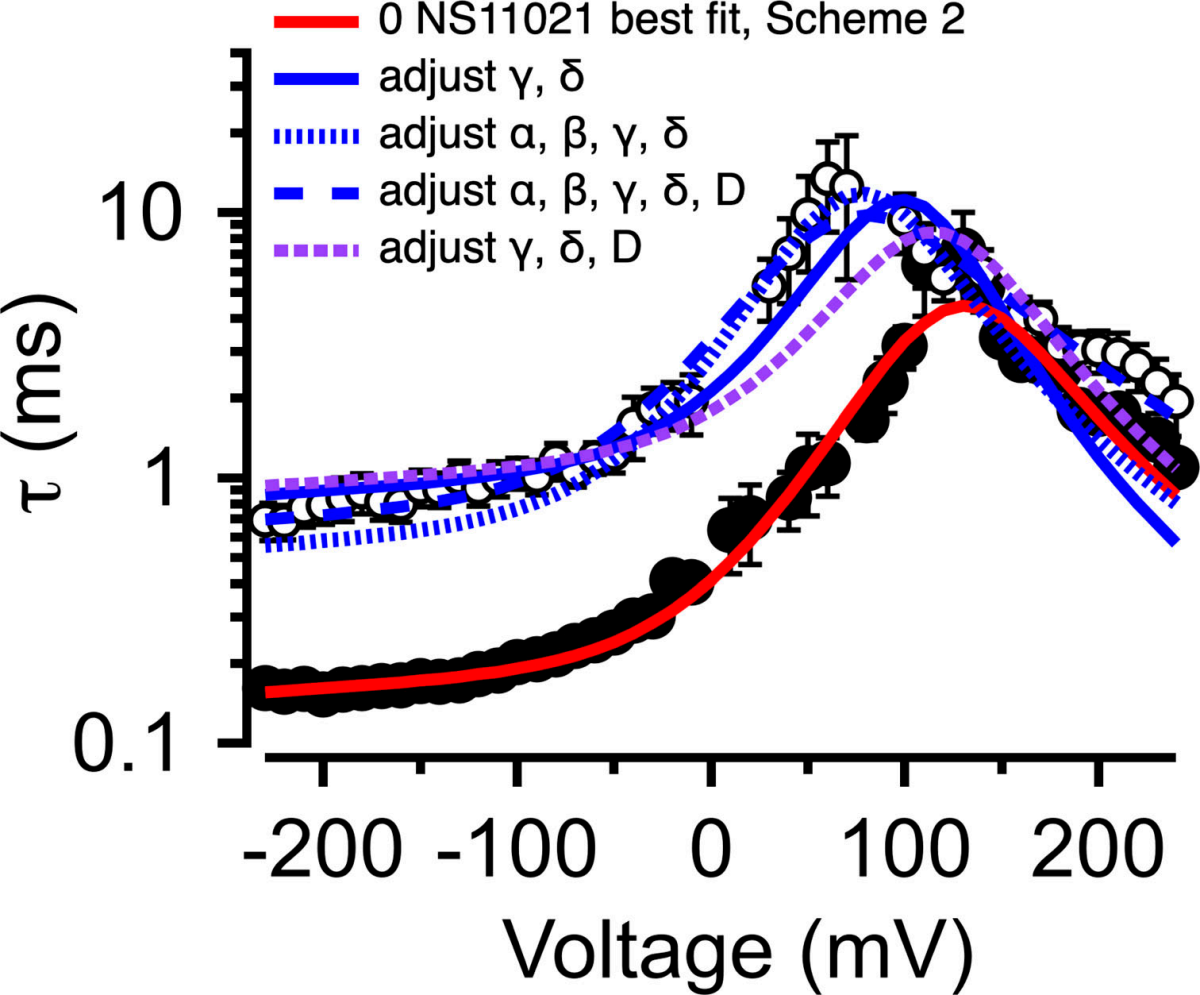
**Description of BK channel activity at nominally 0 Ca^2+^ with 0 or 30 µM NS11021 using** **Scheme 2.** τ versus voltage from patches with nominally 0 µM Ca^2+^, with 0 (filled circles) or 30 µM NS11021 (open circles). Lines represent fits with Scheme 2 using parameters in Table 3 and Table S3: 0 µM NS11021, red; 30 µM NS11021, adjusted for γ δ, solid blue; adjusted for α, β, γ, and δ, dotted blue; adjusted for α, β, γ, δ, and *D*, dashed blue; adjusted for γ, δ, and *D*, dashed purple. These suggest that the major effects of NS11021 on voltage-dependent gating kinetics may be explained in part by adjusting γ and δ in Scheme 2 in combination with adjustments in other voltage-dependent rate constants.

**Figure S9.**
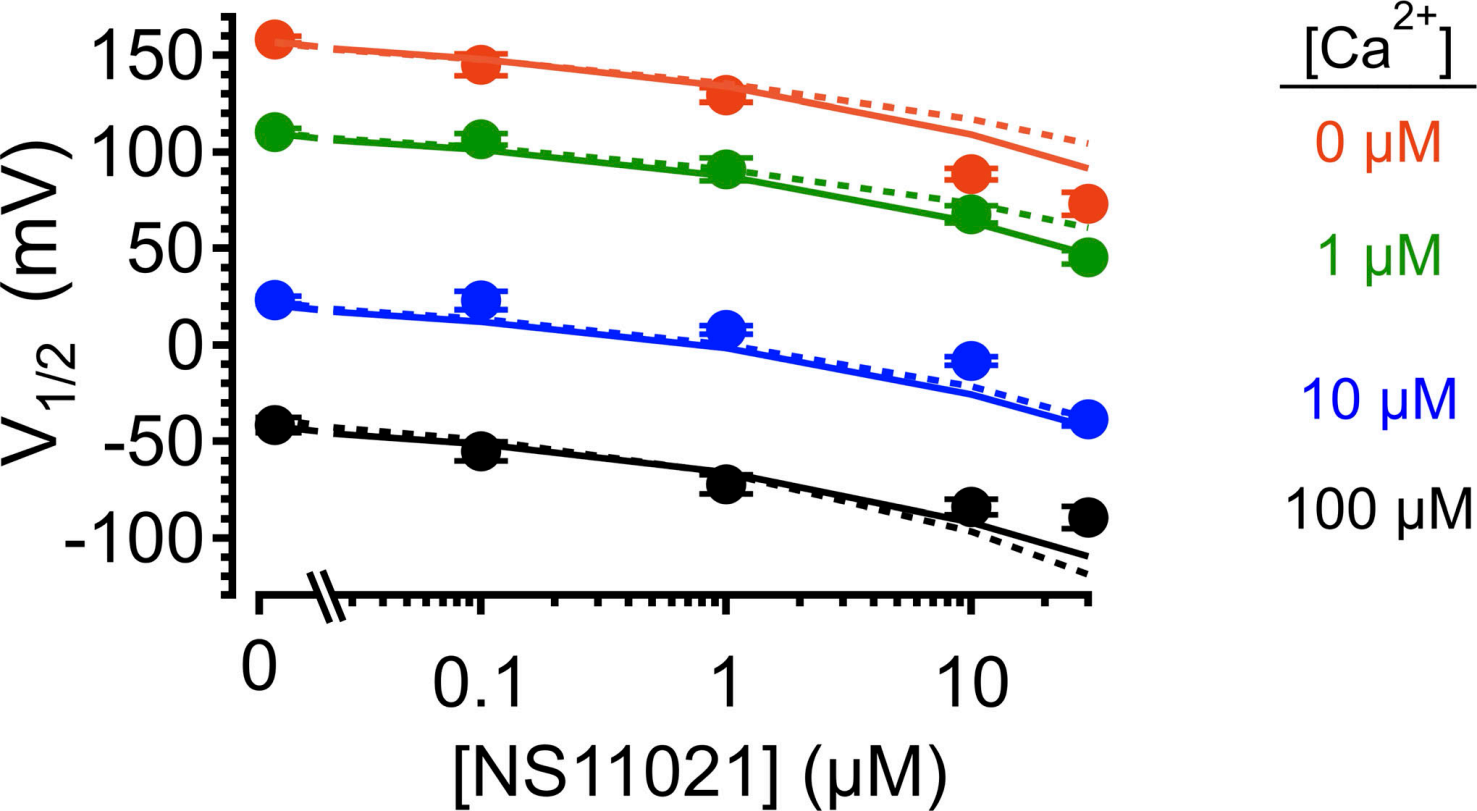
**Effect of NS11021 on *V*_1/2_ over a range of [Ca^2+^].** *V*_1/2_ plotted as a function of [NS11021] for nominally 0 (orange), 1 (green), 10 (blue), and 100 µM Ca^2+^ (black). Experimental data (mean ± SEM, circles) are plotted along with the predicted *V*_1/2_ for Fit A (solid line) and Fit B (dotted line), with adjustable parameter *L*_0_.

**Figure S10.**
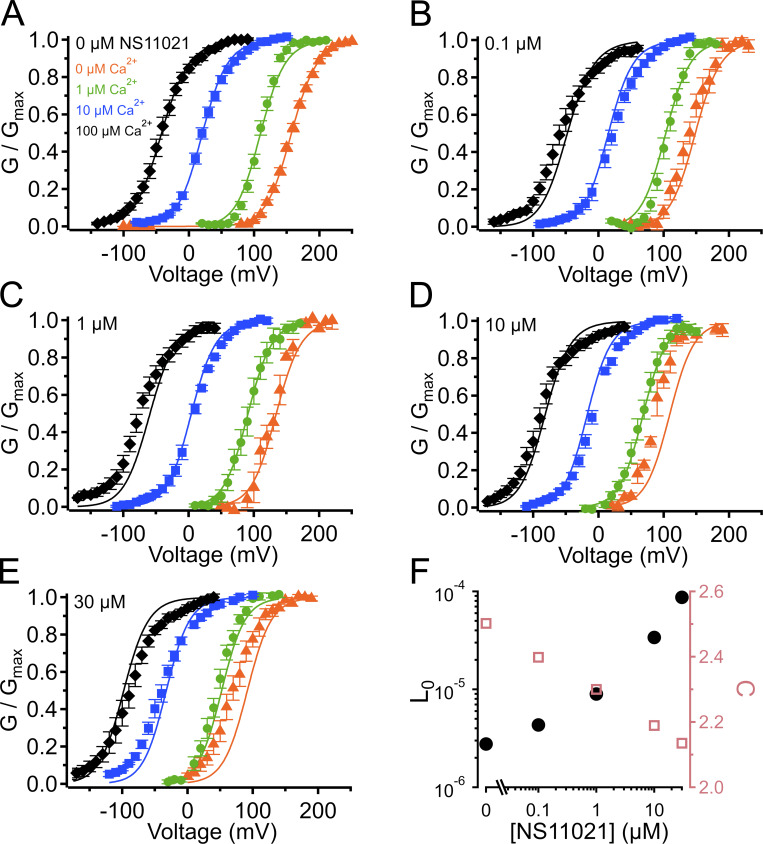
**G–V-Ca^2+^ relations from** **Scheme 1** **using parameters from Fit A to describe changing parameter *C* in addition to *L*_0_.** **(A)** G–V-Ca^2+^ relations in the absence of NS11021 bathed in 0 µM (orange triangle), 1 µM (green circle), 10 µM (blue square), and 100 µM Ca^2+^ (black diamond). Parameters are listed under Fit A in Table 1. Lines represent the predictions with *L*_0_ = 2.8 × 10^−6^, *C* = 2.5. **(B–E)** G–V-Ca^2+^ relations in 0.1, 1, 10, and 30 µM NS11021, respectively. Lines represent predictions with *L*_0_ = 4.3 × 10^−6^, *C* = 2.4 for 0.1 µM NS11021; *L*_0_ = 9.0 × 10^−6^, *C* = 2.3 for 1 µM NS11021; *L*_0_ = 3.4 × 10^−5^, *C* = 2.2 for 10 µM NS11021; *L*_0_ = 8.7 × 10^−5^, *C* = 2.1 for 30 µM NS11021. **(F)** Fitted values for *L*_0_ (black) and C (red) versus [NS11021]. These suggest that the major effects of NS11021 on G–V-Ca^2+^ relations may be explained in part by an increase in *L*_0_ in combination with a decrease in *C*, with increasing [NS11021].

**Figure S11.**
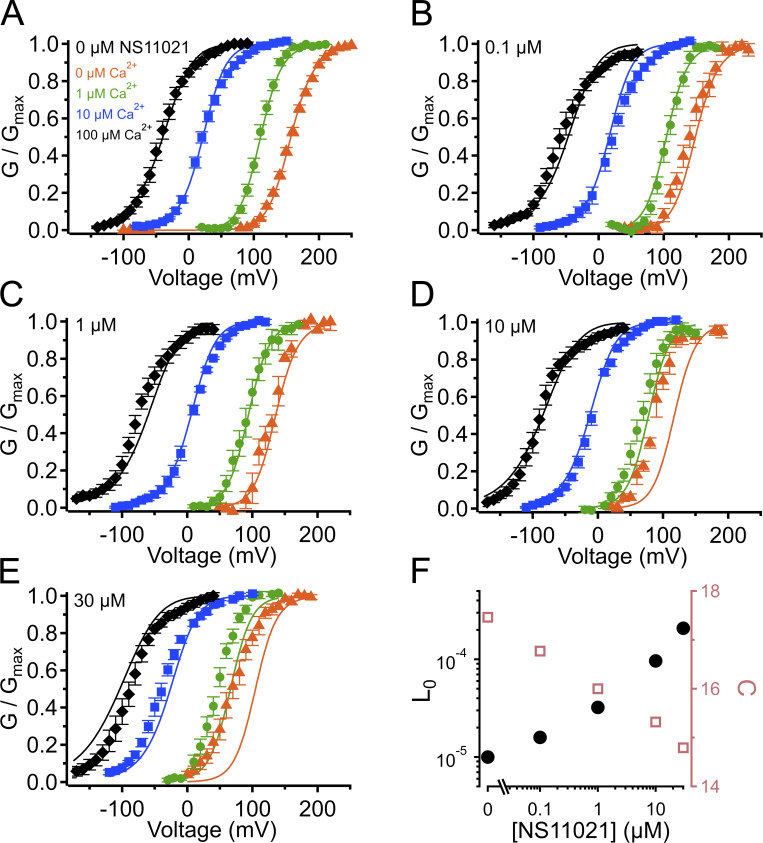
**G–V-Ca^2+^ relations from** **Scheme 1** **using parameters from Fit B to describe changing parameter *C* in addition to *L*_0_.** **(A)** G–V-Ca^2+^ relations in the absence of NS11021 bathed in 0 µM (orange triangle), 1 µM (green circle), 10 µM (blue square), and 100 µM Ca^2+^ (black diamond). Parameters are listed under Fit B in Table 1. Lines represent the predictions with *L*_0_ = 1.0 × 10^−5^, *C* = 18. **(B–E)** G–V-Ca^2+^ relations in 0.1, 1, 10, and 30 µM NS11021, respectively. Lines represent predictions with *L*_0_ = 1.6 × 10^−5^, *C* = 17 for 0.1 µM NS11021; *L*_0_ = 3.2 × 10^−5^, *C* = 16 for 1 µM NS11021; *L*_0_ = 9.6 × 10^−5^, *C* = 15 for 10 µM NS11021; *L*_0_ = 2.1 × 10^−4^, *C* = 15 for 30 µM NS11021. **(F)** Fitted values for *L*_0_ (black) and *C* (red) versus [NS11021]. These suggest that the major effects of NS11021 on G–V-Ca^2+^ relations may be explained in part by an increase in *L*_0_ in combination with a decrease in *C*, with increasing [NS11021].

Table S1 shows mean values of *V*_1/2_ and z determined from Boltzmann fits of individual G–V relations using Eq. 1. Table S2 shows results of changing *L*_0_ plus a second parameter in Scheme 1 to account for effects of NS11021. Table S3 shows additional sets of fitted parameters for Scheme 2 constrained by time constants acquired with nominally 0 Ca^2+^ and either 0 or 30 µM NS11021. Fig. S1 presents representative fits of BK channel activation and deactivation time course with a single exponential for currents with 10 µM Ca^2+^. Fig. S2 shows G–V-Ca^2+^ relations with predictions from Scheme 1 using parameters from Fit A, with adjustable parameter *J*_0_. Fig. S3 shows G–V-Ca^2+^ relations with predictions from Scheme 1 using parameters from Fit A, with adjustable parameter *D*. Fig. S4 shows G–V-Ca^2+^ relations with predictions from Scheme 1 using parameters from Fit B, with adjustable parameter *J*_0_. Fig. S5 shows G–V-Ca^2+^ relations with predictions from Scheme 1 using parameters from Fit B, with adjustable parameter *D*. Fig. S6 shows G–V-Ca^2+^ relations with predictions from Scheme 1 using parameters from Fit B to describe changing parameter *J*_0_ in addition to *L*_0_. Fig. S7 shows G–V-Ca^2+^ relations with predictions from Scheme 1 using parameters from Fit B to describe changing parameter *D* in addition to *L*_0_. Fig. S8 provides a description of BK channel activity at nominally 0 Ca^2+^ with 0 or 30 µM NS11021 using Scheme 2. Fig. S9 shows effects of NS11021 on *V*_1/2_ over a range of [Ca^2+^]. Fig. S10 shows G–V-Ca^2+^ relations with predictions from Scheme 1 using parameters from Fit A to describe changing parameter *C* in addition to *L*_0_. Fig. S11 shows G–V-Ca^2+^ relations with predictions from Scheme 1 using parameters from Fit B to describe changing parameter *C* in addition to *L*_0_.

## Results

### Effects of NS11021 on BK channel activation

**Figure 1.**
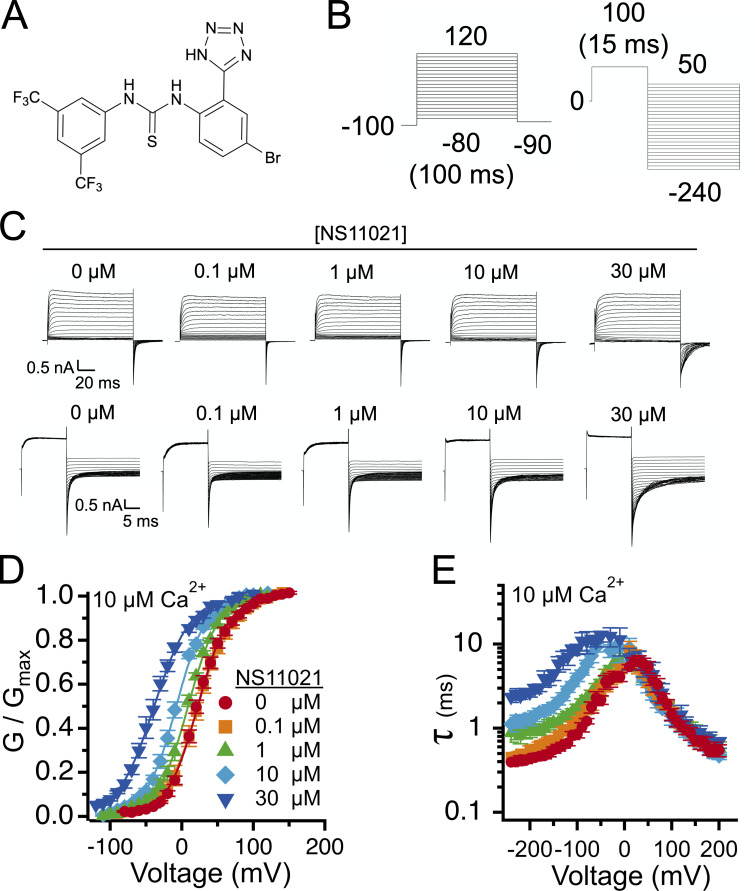
**Effects of NS11021 on BK channel gating.** **(A)** Structure of NS11021. **(B)** Representative voltage protocols for channel activation (left) and deactivation (right). Numbers correspond to voltage (in mV) and duration of activating pulse (in ms). **(C)** Representative BK currents from an inside-out patch with 10 µM Ca^2+^ and the indicated [NS11021] at the cytosolic side of the membrane. “0 µM NS11021” experiments contained 0.1% DMSO (vehicle) with no added NS11021. **(D)** G–V relations from patches with 10 µM Ca^2+^ and the indicated [NS11021]. Solid lines represent fits with a Boltzmann equation. Values for Boltzmann equation parameters are provided in Table S1. **(E)** Time constant of relaxation (τ) versus voltage (10 µM Ca^2+^ and the indicated [NS11021]). Time constants were estimated by fitting activation and deactivation traces with a single exponential (Eq. 2 and Fig. S1). Increasing [NS11021] primarily slows channel deactivation.

To quantify the effect of NS11021 on the voltage dependence of activation, we determined G–V relations from macroscopic recordings of BK currents over a range of [Ca^2+^] and [NS11021] at the cytosolic side of the membrane, from membrane patches excised from HEK-293T cells overexpressing BK channels. Fig. 1 illustrates the effect of NS11021 with 10 µM Ca^2+^ at the cytosolic side of the membrane. We found that increasing [NS11021] results in an increasing leftward shift of the G–V relation, for [NS11021] >0.1 µM. Fitting these G–V relations for channels in the presence of 10 µM Ca^2+^ with Boltzmann equations yielded *V*_1/2_ values (in mV) of 23.4 ± 2.0 (*n* = 20), 23.1 ± 4.6 (*n* = 7), 7.8 ± 2.3 (*n* = 7), −8.2 ± 2.3 (*n* = 6), and −38.5 ± 3.4 (*n* = 6), for 0, 0.1, 1, 10, and 30 µM NS11021, respectively. These leftward shifts in *V*_1/2_ were not associated with changes in the effective gating valence (*z*) of G–V relations. The *z* values for each [NS11021] at 10 µM Ca^2+^ (in e_0_) were 1.29 ± 0.05, 1.13 ± 0.10, 1.22 ± 0.07, 1.22 ± 0.05, and 1.00 ± 0.05, for 0, 0.1, 1, 10, and 30 µM NS11021, respectively. Because the leftward shifting of *V*_1/2_ values does not reach a plateau at the highest [NS11021] used in these experiments, it seems that 30 µM NS11021 is not sufficient to produce a maximal effect on the channel. However, the limited solubility of the drug in aqueous solutions posed a barrier to measurements at [NS11021] >30 µM. Nevertheless, under these conditions, increasing [NS11021] substantially activates BK channels. Based on our current understanding of BK channel gating, the leftward shifts in *V*_1/2_ observed with 10 µM Ca^2+^ could arise through the drug acting at the VSD, CSD, or PGD or at positions that effect energetic coupling between domains, mechanisms that will be explored below.

### NS11021 slows channel deactivation time course with little effect on activation

The leftward shift in G–V relation could be attributed to an action of NS11021 on voltage-dependent BK channel activation, deactivation, or both. To gain further insight toward the underlying mechanism, we analyzed the time course of channel activation and deactivation as a function of voltage over a range of [NS11021], using increasing depolarizing voltage steps applied to channels held in the closed state, and increasing hyperpolarizing steps applied to channels held in the open state, respectively (Fig. 1, B and C). As observed previously, macroscopic BK current kinetics were observed to be essentially monoexponential, and currents were fitted directly with single exponential functions to estimate activation or deactivation time constant (τ; Fig. S1; Horrigan et al., 1999; Cui et al., 1997).

At 10 µM Ca^2+^, we observed that increasing [NS11021] resulted primarily in marked slowing of tail current decays, which was apparent at voltages more negative than the activation *V*_1/2_ (Fig. 1 C, top traces). Time constants of BK current relaxation determined by exponential fitting were plotted as a function of voltage for each [NS11021] (Fig. 1 E). From these data, it was apparent that increasing [NS11021] elicited a marked slowing of channel deactivation of a range of hyperpolarizing voltages. For channel deactivation, the value for τ (in ms) at −200 mV in 10 µM Ca^2+^ for each [NS11021] was 0 µM = 0.44 ± 0.04 (*n* = 9), 0.1 µM = 0.55 ± 0.07 (*n* = 7), 1 µM = 0.92 ± 0.15 (*n* = 5), 10 µM = 1.29 ± 0.22 (*n* = 4), and 30 µM = 2.66 ± 0.31 (*n* = 5).

In contrast, time constants for channel activation measured at depolarized voltages were affected relatively little with increasing [NS11021]. The value for τ (in ms) at +200 mV in 10 µM Ca^2+^ for each [NS11021] was 0 µM = 0.55 ± 0.09, 0.1 µM = 0.79 ± 0.23, 1 µM = 0.59 ± 0.10, 10 µM = 0.47 ± 0.04, and 30 µM = 0.68 ± 0.10. These results suggest a mechanism in which NS11021 acts on a region of the channel that underlies a distinct subset of transitions in the gating pathway.

**Figure 2.**
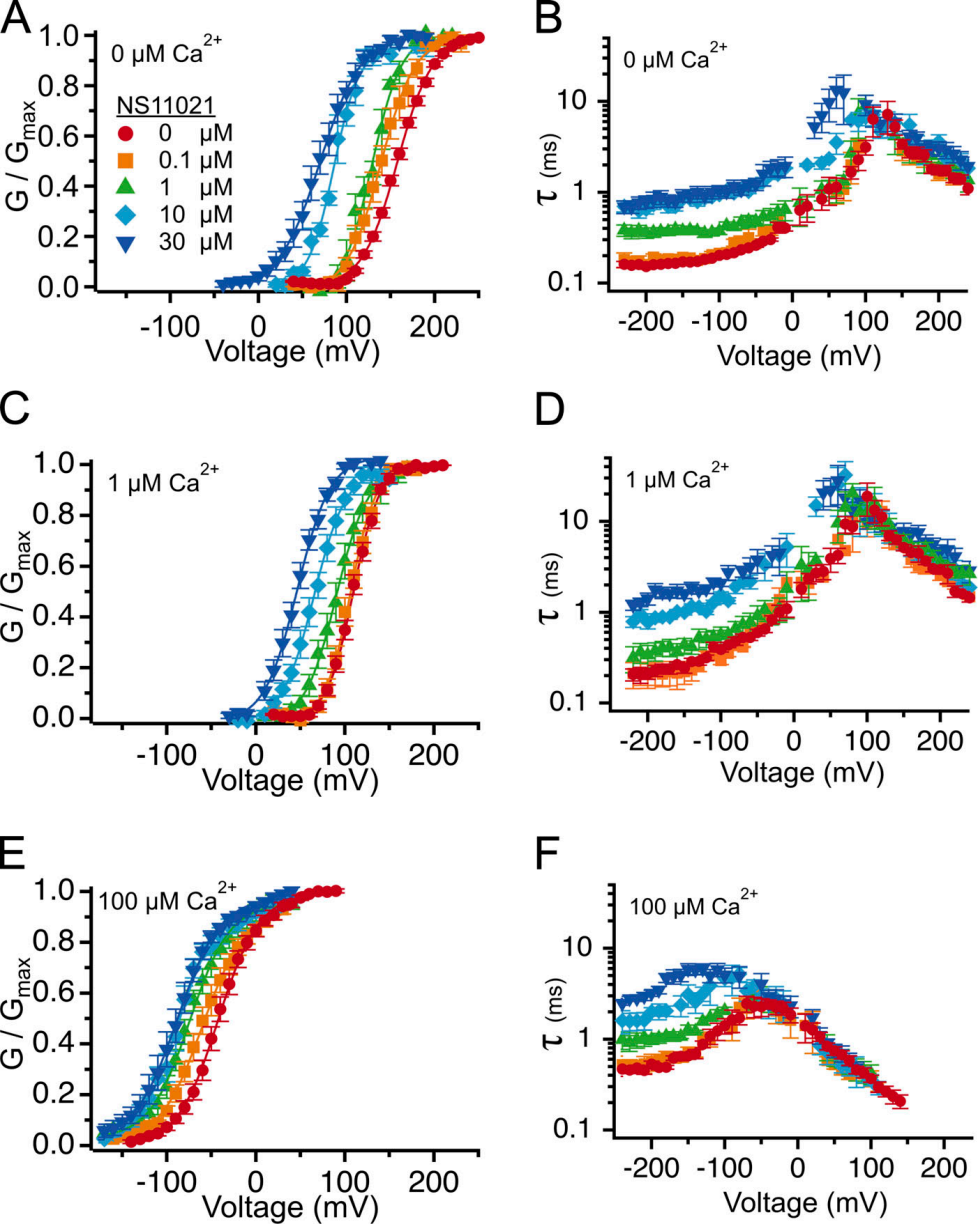
**NS11021 activates BK channels over a range of [Ca^2+^].** **(A)** G–V relations from patches with nominally 0 µM Ca^2+^. **(B)** Time constant (τ) versus voltage from patches with nominally 0 µM Ca^2+^. Time constant at −200 mV (τ_−__200_) for each [NS11021]: 0 µM, 0.15 ± 0.01 ms (*n* = 33); 0.1 µM, 0.16 ± 0.02 ms (*n* = 11); 1 µM = 0.36 ± 0.05 ms (*n* = 7); 10 µM, 0.65 ± 0.08 ms (*n* = 5); 30 µM, 0.79 ± 0.12 ms (*n* = 14). **(C)** G–V relations from patches with 1 µM Ca^2+^. **(D)** τ versus voltage from patches with 1 µM Ca^2+^. τ_−__200_ for each [NS11021]: 0 µM, 0.21 ± 0.03 ms (*n* = 21); 0.1 µM, 0.20 ± 0.06 ms (*n* = 8); 1 µM, 0.34 ± 0.09 ms (*n* = 10); 10 µM, 0.78 ± 0.12 ms (*n* = 7); 30 µM, 1.41 ± 0.21 ms (*n* = 11). **(E)** G–V relations from patches with 100 µM Ca^2+^. **(F)** τ versus voltage from patches with 100 µM Ca^2+^. τ_−__200_ for each [NS11021]: 0 µM, 0.53 ± 0.04 ms (*n* = 11); 0.1 µM, 0.56 ± 0.12 ms (*n* = 8); 1 µM, 0.99 ± 0.11 ms (*n* = 6); 10 µM, 2.01 ± 0.22 ms (*n* = 6); 30 µM = 3.22 ± 0.25 ms (*n* = 9). In A, C, and E, solid lines represent fits with a Boltzmann equation; parameters can be found in Table S1.

The results with 10 µM Ca^2+^ establish benchmarks for further analysis of BK channel activation by NS11021. To determine the relation between the effects of NS11021 and activation of the CSD, we measured G–V and τ versus *V* relations for BK channels over a range of [NS11021] at several [Ca^2+^], ranging from nominally 0 Ca^2+^ (buffered with 5 mM EGTA) to 100 µM Ca^2+^. Fig. 2 illustrates that under each condition of 0 through 100 µM Ca^2+^, increasing [NS11021] yields qualitatively similar effects, namely a leftward shift in activation *V*_1/2_ and slowing of time constants for voltage-dependent deactivation, but essentially no NS11021-dependent change in voltage-dependent activation time course.

The observation that NS11021 elicits similar dose-dependent shifts in activation *V*_1/2_ and slowing of deactivation time constants at nominally 0 Ca^2+^ as it does with higher Ca^2+^ is consistent with the ideas that (1) NS11021 does not act primarily through CSD activation, and (2) NS11021 is not Ca^2+^-mimetic. If one compares the action of NS11021 at any single [Ca^2+^] versus the action of Ca^2+^ at any single [NS11021], one can see that increasing [Ca^2+^] results in hyperpolarizing shifts in *V*_1/2_ that are associated with both speeding up of the activation time course and slowing of the deactivation time course, actions that together are distinct from that of NS11021. In addition, the asymptotic slowing of deactivation kinetics at very hyperpolarized voltages (less than −200 mV) over the entire examined range of [Ca^2+^] with increasing NS11021 is consistent with the idea that NS11021 is acting on BK channels when VSDs are in the resting state.

### NS11021 activation does not require the CSD

**Figure 3.**
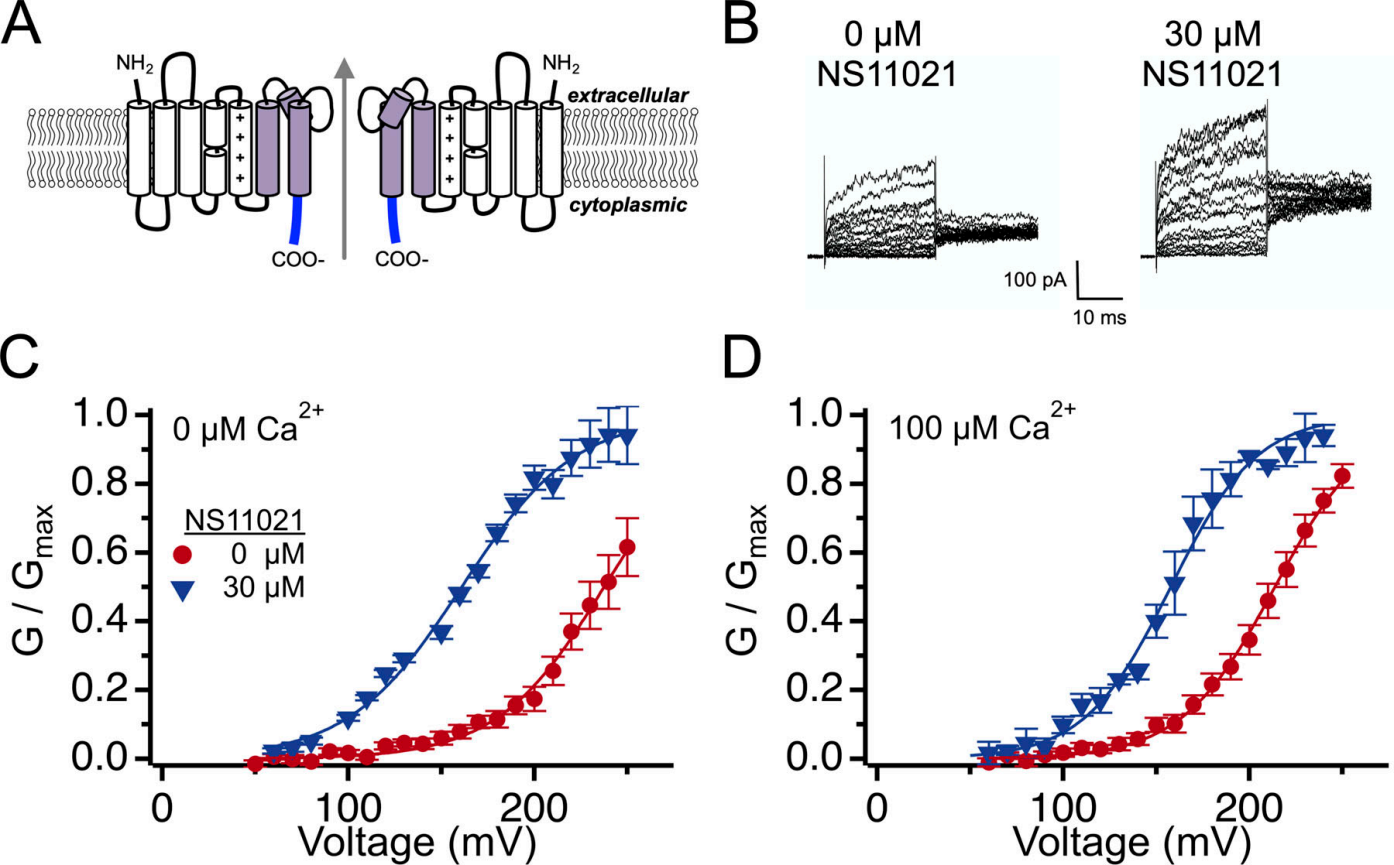
**Effects of NS11021 on truncated BK channels.** **(A)** Topology of the truncated BK channel construct Slo1c-Kv-MinT, showing two of the four domains side by side. Arrow illustrates the permeation pathway. The VSD (S0–S4, white), PGD (S5–S6, purple), and 11 residues from the Kv1.4 tail (blue) are sufficient for trafficking of these voltage-gated channels to the plasma membrane. **(B)** Representative Slo1c-Kv-MinT currents (nominally 0 Ca^2+^). Patches were held at 0 mV and stepped to voltages ranging from +50 mV to +250 mV, followed by a step to +160 mV for tail current measurement. **(C)** G–V relations from Slo1c-Kv-MinT at nominally 0 Ca^2+^, with 0 (red) or 30 µM NS11021 (blue). *V*_1/2_ shifted from 238 ± 8.1 mV (*n* = 13) to 161 ± 2.6 mV (*n* = 7) with addition of 30 µM NS11021. **(D)** G–V relations from Slo1c-Kv-MinT at 100 µM Ca^2+^, with 0 (red) or 30 µM NS11021 (blue). For these data, *V*_1/2_ shifted from 214 ± 3.4 mV (*n* = 24) to 158 ± 2.0 mV (*n* = 5) with addition of 30 µM NS11021.

Although the activating effect of NS11021 on BK channels in nominally 0 Ca^2+^ suggests that the action of NS11021 does not require CSD activation, we hypothesized that the CSD could yet play a structural role in NS11021 action, perhaps through binding at the CSD–VSD interface. To test this, we assayed the effect of NS11021 in BK channels lacking the CSD, using the construct Slo1C-Kv-minT (Fig. 3 A; Budelli et al., 2013). In this construct, the BK channel is truncated at the end of the C-linker following the PGD (at residue R342), and the CSD is replaced by an 11-residue sequence derived from the C-terminal region of the Kv1.4 channel, to enable its trafficking to the plasma membrane. This results in a channel with voltage-activation properties similar to WT BK channels in nominally 0 Ca^2+^, whereas the absence of the CSD renders the channel nonresponsive to Ca^2+^.

Fig. 3 illustrates that in nominally 0 Ca^2+^, voltage-dependent activation of Slo1C-Kv-minT channels is shifted toward hyperpolarizing voltages by NS11021. We evaluate these Slo1C-Kv-minT recordings with caution, as the requirement for very depolarized activating voltages (≥200 mV) combined with relatively low macroscopic current amplitudes (∼100 pA) contributed to experimental variability. Nonetheless, we observed that the 75-mV shift elicited by 30 µM NS11021 to Slo1C-Kv-minT channels was not significantly different from the ∼85-mV hyperpolarizing shift elicited by 30 μM NS11021 in WT BK channels in nominally 0 Ca^2+^ (P = 0.43, two-tailed *t* test), consistent with the idea that NS11021 activation does not require the presence of the CSD. Additionally, we observed that voltage activation of Slo1C-Kv-minT channels is not substantially affected by the presence of 100 µM Ca^2+^ at the cytosolic side of the patch, and under these conditions 30 µM NS11021 elicited a similar shift in *V*_1/2_ to 158 mV. Together, these results support the idea that action of NS11021 does not absolutely require CSD activation and further suggest that presence of the CSD is not a structural requirement for NS11021 binding or activation.

### NS11021 activation may occur primarily through the PGD

**Figure 4.**
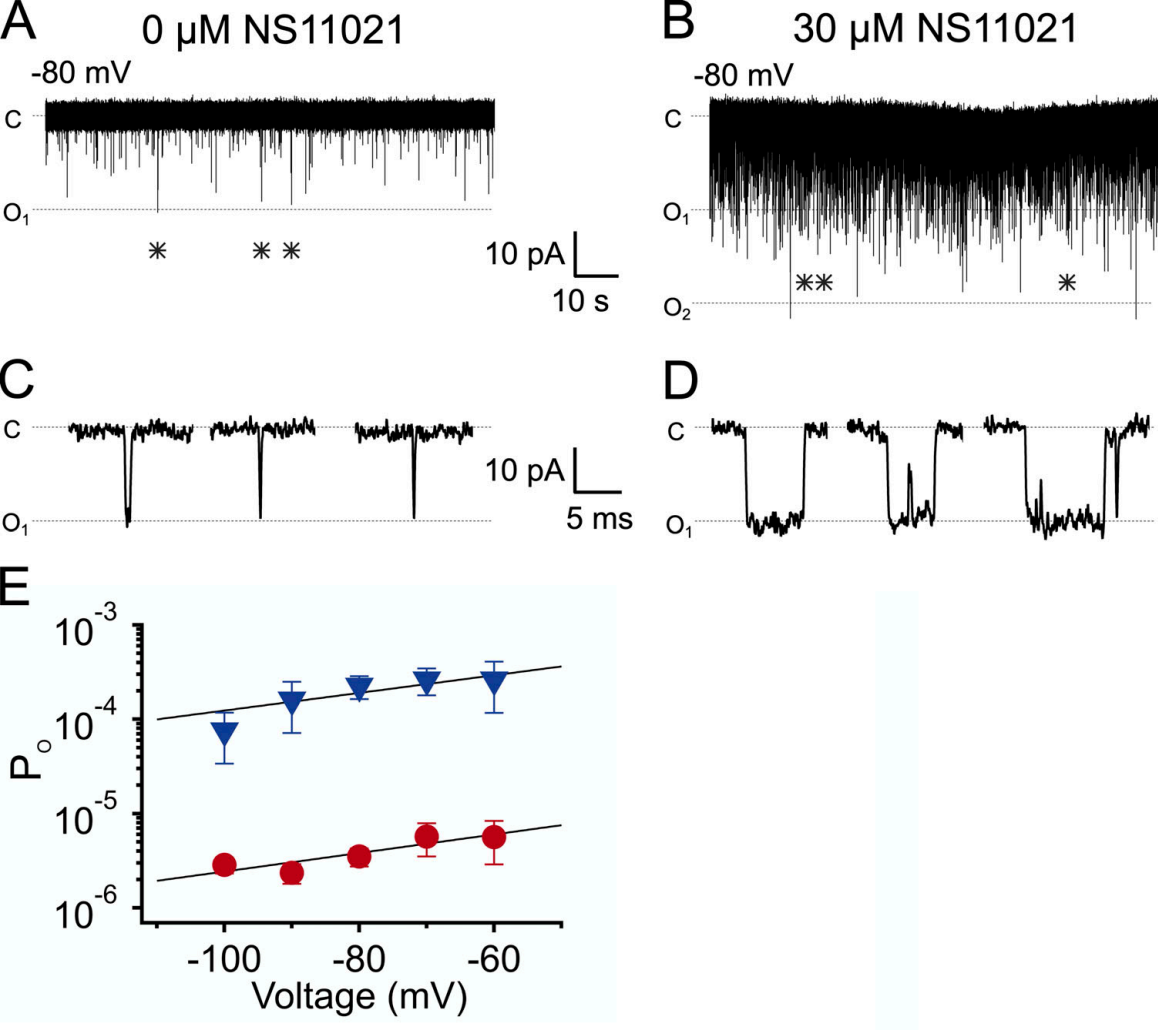
**NS11021 increases P_o_ at negative voltages in nominally 0 Ca^2+^.** **(A)** Representative BK currents in nominally 0 Ca^2+^ at −80 mV, with 0 µM NS11021. NP_o_ = 2.5 × 10^−4^. **(B)** BK currents from the same patch as in A, following addition of 30 µM NS11021. NP_o_ = 1.5 × 10^−2^. Patch was estimated to have 127 active channels, determined by dividing the maximum macroscopic current amplitude by the single-channel conductance. **(C and D)** Representative channel openings from the traces in A and B, respectively (from positions indicated by *), at an expanded time scale. **(E)** P_o_ versus voltage for 0 µM (red circles) and 30 µM NS11021 (blue triangles) activity. Data points represent mean P_o_ ± SEM from three to eight experiments. Lines represent best fit with P_o_ = *L*_0_exp(−*z_L_V*/*k_B_T*). Parameters were 0 µM NS11021 (*z_L_* = 0.57 e_0_, *L*_0_ = 2.3 × 10^−5^); 30 µM NS11021 (*z_L_* = 0.55 e_0_, *L*_0_ = 1.1 × 10^−3^). Estimated numbers of channels in these multichannel patches ranged from 22 to 199.

If NS11021 action does not require the CSD, then we reasoned that it may occur through the VSD, PGD, and/or through an effect on VSD–PGD coupling. To distinguish among these possibilities, we quantified BK channel gating in nominally 0 Ca^2+^ at negative voltages (−60 mV or less), to drive the channel toward open and closed conformations in which both the CSD and VSD are largely at rest. Under these conditions, BK channels gate with very low P_o_, such that single-channel openings can be resolved in patches containing many (∼20–200) active channels (Fig. 4, A–D; Koval et al., 2007; Horrigan and Aldrich, 2002; Wang et al., 2009, 2006). We observed that in the absence of NS11021, BK channels gated with a P_o_ of (3.5 ± 0.8) × 10^−6^ (*n* = 8) at −80 mV, and addition of 30 µM NS11021 increased the P_o_ by 62-fold to (2.2 ± 0.6) × 10^−4^ (*n* = 8). This result is consistent with the idea that NS11021 action does not require either CSD or VSD activation and may thus occur largely through an effect at the PGD.

**Figure 5.**
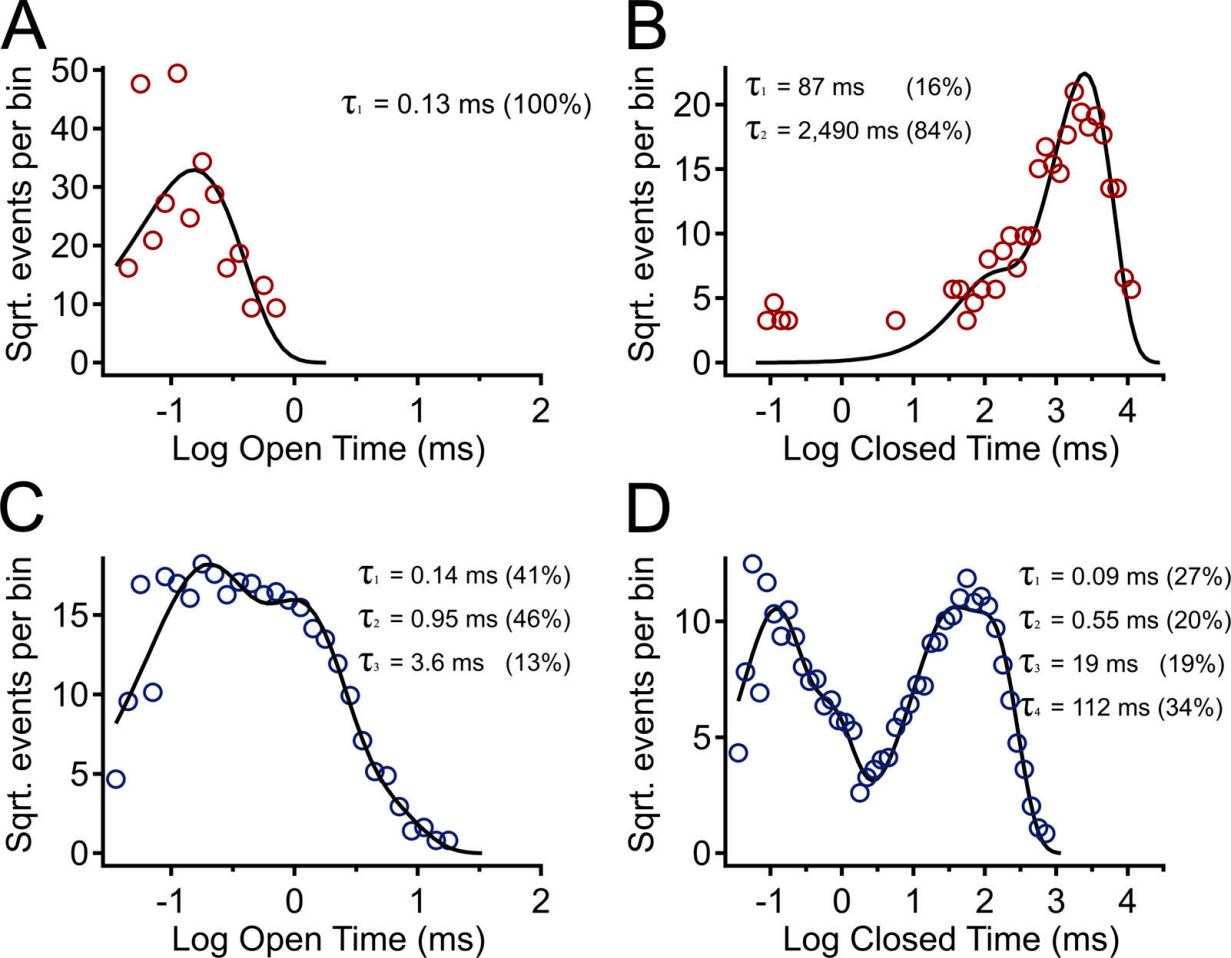
**Effects of NS11021 on open and closed dwell time distributions.** **(A)** Distribution of open times with 0 µM NS11021 (red circles). Line represents fit with a single exponential, τ = 0.13 ms. Distribution contains 396 events; this and subsequent distributions were normalized to contain 100,000 events. **(B)** Distribution of closed times with 0 µM NS11021 (red circles). Line represents fit with sum of two exponentials, with τ and percentage area of each component as indicated. Number of events = 395. **(C)** Distribution of open times with 30 µM NS11021 (blue circles) added to the same patch. Line represents fit with sum of three exponentials, with τ and percentage of area as indicated. Number of events = 11,115. **(D)** Distribution of closed times with 30 µM NS11021 (blue circles). Line represents fit with sum of four exponentials, with τ and percentage area as indicated. Number of events = 11,145. Distributions in A–D show activity in nominally 0 Ca^2+^, −80 mV.

Under these conditions, it was possible to directly measure the durations of BK channel openings; with nominally 0 Ca^2+^ and at −80 mV, the mean open time was 0.15 ± 0.01 ms (*n* = 5), and this increased to 0.52 ± 0.07 ms (*n* = 5) with addition of 30 µM NS11021 (Fig. 5, A and C). This increase in mean open time was associated with a relative increase in the frequency of openings (i.e., a decrease in mean closed time), although mean closed times are sensitive to the number of channels in the patch. To gain further insight toward NS11021 actions on channel gating, we constructed open dwell time distributions (Fig. 5, A and C). At −80 mV, open times were described by a single exponential component with a time constant of 0.140 ± 0.004 ms (*n* = 5). In the presence of 30 µM NS11021, open times were best described by three components, with τ_1_ = 0.09 ± 0.01 ms (area = 0.41 ± 0.02), τ_2_ = 0.53 ± 0.09 (area = 0.39 ± 0.02), and τ_3_ = 2.5 ± 0.4 ms (area = 0.20 ± 0.02). Thus, NS11021 drives the channel to gate among additional long lifetime open states.

To gain further insight toward channel gating under conditions where the CSD and VSD are at rest, we compared closed dwell time distributions with 0 and 30 µM NS11021 (Fig. 5, B and D). Whereas the number of active channels in the patch determines the closed times, Fig. 5, B and D, show distributions constructed from measurements performed on the same patch; thus, effects of NS11021 on these dwell times can be determined from a direct comparison. These show a >20-fold decrease in the time constant for the long-closed component (from 2,490 to 112 ms). In addition, the fraction of closings with lifetimes <1 ms comprised few closings in 0 µM NS11021, whereas this increased to 47% of closings with 30 µM NS11021 (similar results were observed in four additional patches). Because the majority of these brief closings are likely found within bursts of openings, these results support the idea that NS11021 drives BK channel gating from single, brief openings (Fig. 4 C) toward bursts of two or more longer openings (Fig. 4 D) under conditions where the VSD and CSD are presumably at rest.

### Concentration dependence of NS11021 action

NS11021 activates BK channels over a wide range of Ca^2+^ and voltages, through a mechanism that may not involve the CSD or VSD modules. Under the conditions of our experiments, these channels are presumed to be homotetramers. If NS11021 were found to act at the VSD or CSD, then it might be reasonable to assume a minimal stoichiometry of four NS11021 molecules binding to activate one BK channel. However, an action at the PGD, located at the confluence of the four subunits, might require fewer than four NS11021 molecules; for example, binding of one or two NS11021 molecules might hinder binding of additional molecules. Alternatively, binding of fewer than four NS11021 molecules may be sufficient to produce a maximal effect, or multiple NS11021 molecules may act in a noncooperative manner on each subunit to stabilize the open state. To gain further insight toward the drug activation mechanism, we analyzed the relation between [NS11021] and channel activity by fitting *G*/*G*_max_ data at individual voltages over a range of [NS11021] with a Hill equation. To include the widest range of conditions in our analysis, we examined all voltages at which the minimum *G*/*G*_max_ (at 0 µM NS11021) was <0.5. Although strictly empirical, this analysis can be used as an indicator of the minimal stoichiometry for drug–receptor interaction, and in the case of multiple binding sites, whether the ligand exhibits either positive or negative cooperativity (i.e., increases or decreases in coupling energetics when multiple sites are occupied).

**Figure 6.**
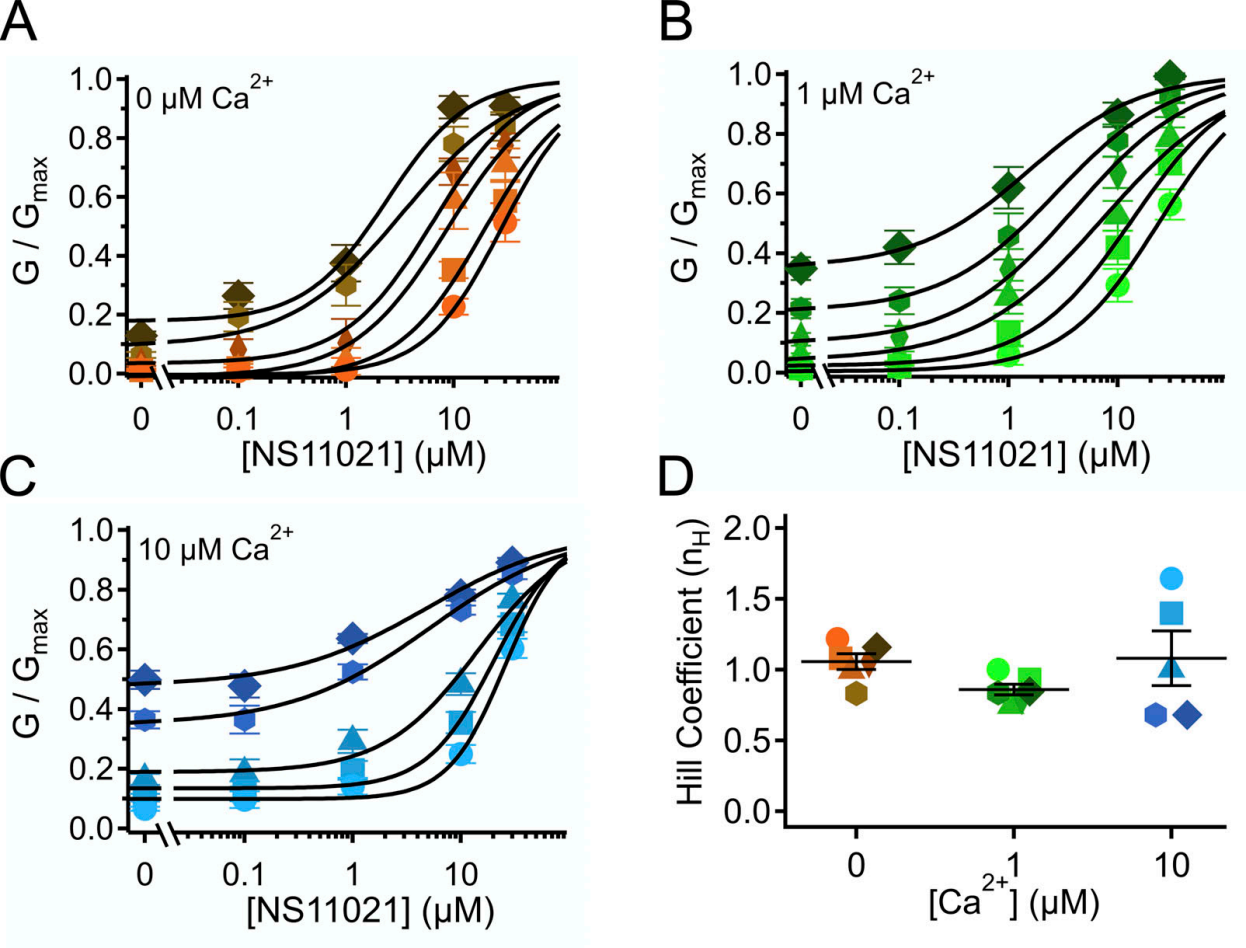
**Estimate of apparent stoichiometry of NS11021 action.** **(A)** Normalized G (*G*/*G*_max_) versus [NS11021] in nominally 0 Ca^2+^. Data were grouped by voltage and fitted with a Hill equation (Eq. 3; black lines) to estimate Hill coefficient (*n_H_*) and EC_50_. Parameters at each voltage were 70 mV (circle; *n_H_* = 1.2; EC_50_ = 28 µM), 80 mV (square; *n_H_* = 1.1; EC_50_ = 20 µM), 90 mV (triangle; *n_H_* = 1.0; EC_50_ = 8.8 µM), 100 mV (diamond; *n_H_* = 1.1; EC_50_ = 6.4 µM), 110 mV (hexagon; *n_H_* = 0.8; EC_50_ = 3.4 µM), and 120 mV (tilted square; *n_H_* = 1.2; EC_50_ = 2.4 µM). **(B)** *G*/*G*_max_ versus [NS11021] with 1 µM Ca^2+^. 50 mV (circle; *n_H_* = 1.0; EC_50_ = 24 µM), 60 mV (square; *n_H_* = 0.9; EC_50_ = 13 µM), 70 mV (triangle; *n_H_* = 0.7; EC_50_ = 7.5 µM), 80 mV (diamond; *n_H_* = 0.8; EC_50_ = 4.0 µM), 90 mV (hexagon; *n_H_* = 0.8; EC_50_ = 2.7 µM), and 100 mV (tilted square; *n_H_* = 0.8; EC_50_ = 1.5 µM). **(C)** *G*/*G*_max_ versus [NS11021] with 10 µM Ca^2+^. −30 mV (circle; *n_H_* = 1.6; EC_50_ = 26 µM), −20 mV (square; *n_H_* = 1.4; EC_50_ = 21 µM), −10 mV (triangle; *n_H_* = 1.0; EC_50_ = 14 µM), 10 mV (hexagon; *n_H_* = 0.7; EC_50_ = 5.3 µM), and 20 mV (tilted square; *n_H_* = 0.7; EC_50_ = 4.9 µM). **(D)** Hill coefficients at each [Ca^2+^]; parameters at each voltage are shown as circles; mean at a given [Ca^2+^] is indicated by horizontal line, and error bars show the SEM. Means for each [Ca^2+^] are 0 Ca^2+^ (1.06 ± 0.06; *n *= 6), 1 µM Ca^2+^ (0.86 ± 0.04; *n* = 6), and 10 µM Ca^2+^ (1.08 ± 0.19; *n* = 5).

Fig. 6 illustrates that over the range of [Ca^2+^] examined (including at nominally 0 Ca^2+^), *G*/*G*_max_ versus [NS11021] relations at individual voltages could be described with Hill coefficients close to 1. To a first approximation, we observed no apparent correlation between the Hill coefficient steepness and the voltage at which it was determined. This was complicated by the observation that at 10 µM Ca^2+^, we included dose–response relations at depolarized voltages where *G*/*G*_max_ at 0 µM NS11021 was close to 0.5, which yielded slightly more shallow Hill coefficients.

Although these estimates do not confirm that there is an obligatory 1:1 relation for NS11021 to act on the BK channel, they do effectively rule out the idea that multiple NS11021 molecules activate the channel through a highly positive cooperative mechanism and are consistent with mechanisms in which either a single NS11021 molecule is sufficient to activate the channel, or multiple NS11021 molecules stabilize activated channel subunits effectively independently.

### NS11021 acts by stabilizing the PGD in the open state

To gain further insight toward the molecular mechanism of NS11021 action, we developed a quantitative description of gating in the presence and absence of drug, based on the well-established dual allosteric model for BK channel gating Scheme 1. Although BK channels are known to contain a total of eight high-affinity Ca^2+^ binding sites per channel, here we have used a simplified gating scheme containing only four Ca^2+^ binding sites per channel and four voltage sensors that are each activated independent of one another in single transitions (Rothberg and Magleby, 1999; Horrigan and Aldrich, 2002). Our approach was to first identify a set of parameters to describe the steady-state G–V relations over a range of [Ca^2+^] (G–V-Ca^2+^ relations), which would be constrained by our experimental single-channel and macroscopic current data. Because some parameters in the kinetic scheme are poorly constrained by these data, we developed two sets of parameters, Fit A and Fit B, which yielded nearly equivalent descriptions of the data as evaluated by χ^2^ statistic (see Materials and methods and Table 1). After identifying these sets of parameters, we next evaluated whether the G–V-Ca^2+^ relations in the presence of increasing [NS11021] could be described by changing only a single parameter, while keeping the other parameters at their values determined from fits with data in the absence of NS11021. Thus, we attempted fits by varying only *J*_0_, *K*, *L*_0_, etc., to determine which parameter [NS11021] had the single greatest impact, also evaluated by a χ^2^ statistic.

**Table 1.** Parameters for Scheme 1, fitted with data at 0 µM NS11021

Parameter	Fit A	Fit B
*J*_0_	0.08	0.06
*K_D_* (µM)	41	17
*L*_0_	2.8 × 10^−6^	1.0 × 10^−5^
*z_J_* (e_0_)	0.30	0.41
*z_L_* (e_0_)	0.57	0.58
*C*	2.5	17
*D*	29	16
*E*	20	1.1

**Figure 7.**
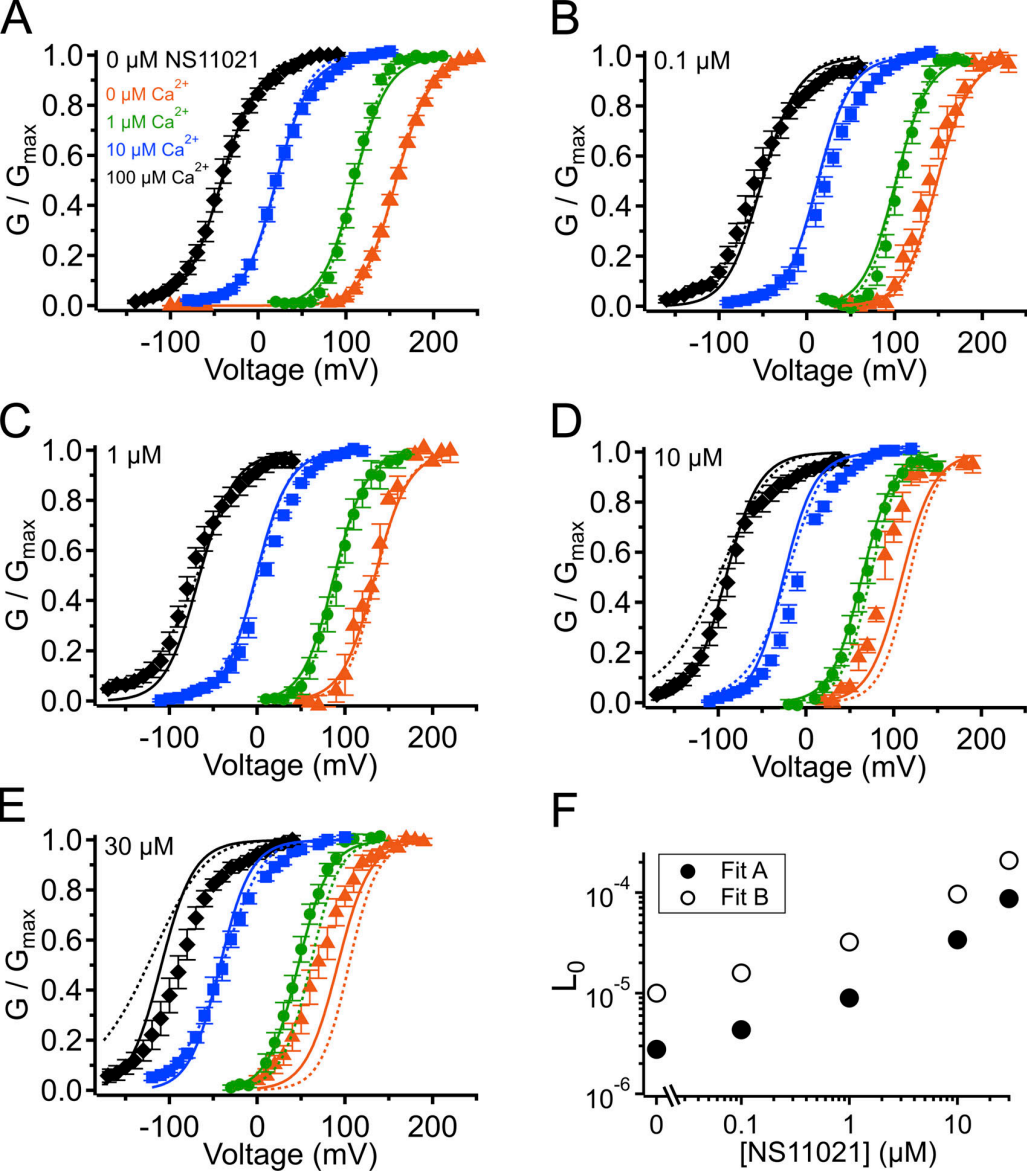
**G–V-Ca^2+^ relations with predictions from** **Scheme 1** **using parameters from Fits A and B.** **(A)** G–V-Ca^2+^ relations in the absence of NS11021 bathed in 0 µM (orange triangle), 1 µM (green circle), 10 µM (blue square), and 100 µM Ca^2+^ (black diamond). Parameters are listed in Table 1. Lines represent the predictions with *L*_0_ = 2.8 × 10^−6^ (Fit A, solid lines) and *L*_0_ = 1.0 × 10^−5^ (Fit B, dashed lines). **(B–E)** G–V-Ca^2+^ relations in 0.1, 1, 10, and 30 µM NS11021, respectively. Lines represent predictions with *L*_0_ = 4.3 × 10^−6^ (0.1 µM), 9.0 × 10^−6^ (1 µM), 3.4 × 10^−5^ (10 µM), and 8.7 × 10^−5^ (30 µM) for Fit A, and *L*_0_ = 1.6 × 10^−5^ (0.1 µM), 3.2 × 10^−5^ (1 µM), 9.6 × 10^−5^ (10 µM), and 2.1 × 10^−4^ (30 µM) for Fit B. **(F)** Fitted values for *L*_0_ versus [NS11021] (Fit A as black/filled circles, Fit B as white/open circles). These suggest that the major effects of NS11021 on G–V-Ca^2+^ relations may be explained in part by an increase in *L*_0_ with increasing [NS11021].

**Figure 8.**
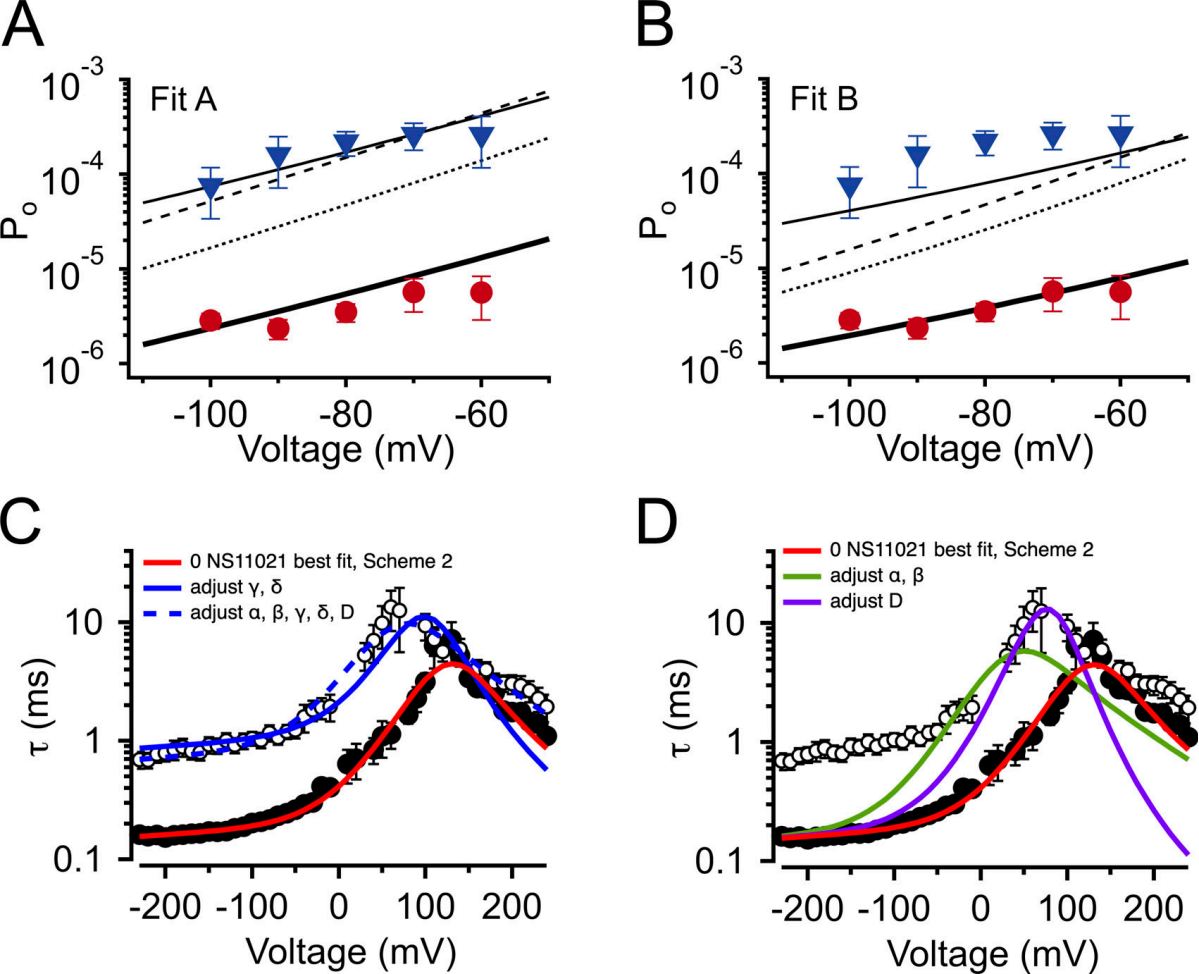
**Description of BK channel activity at nominally 0 Ca^2+^ using** **Schemes 1 and 2.** **(A)** P_o_ versus voltage with 0 μM NS11021 (red circles) and 30 µM NS11021 (blue triangles). Solid line represents P_o_ predicted from Scheme 1 using parameters in Table 1, Fit A. Predicted P_o_ with 30 µM NS11021 was generated by using Fit A substituting *L*_0_ = 8.7 × 10^−5^ (solid thin line), *J*_0_ = 0.32 (dashed line), or *D* = 74 (dotted line). Using parameters from Fit A in Table 1 resulted in a χ^2^ value of 3.85. χ^2^ values obtained by changing only one parameter to account for P_o_ at 30 µM NS11021: for *L*_0_, 0.41; for *J*_0_, 0.77; for *D*, 3.15; thus, substitution of *L*_0_ resulted in the lowest χ^2^ value (best fit) for these data. **(B)** P_o_ versus voltage as in A, except with solid line showing P_o_ predicted using Fit B. P_o_ with 30 µM NS11021 was generated with Fit B by substituting *L*_0_ = 2.1 × 10^−4^ (solid thin line), *J*_0_ = 0.36 (dashed line), or *D* = 61 (dotted line). Using parameters from Fit B resulted in a χ^2^ value of 1.18. χ^2^ values obtained by changing only one parameter to account for P_o_ at 30 µM NS11021: for *L*_0_, 1.91; for *J*_0_, 3.13; for *D*, 4.73. Again, substitution of *L*_0_ resulted in the lowest χ^2^ value (best fit) for these data. All χ^2^ values for A and B based on five total data points. **(C)** τ versus voltage from patches with nominally 0 µM Ca^2+^, with 0 (filled circles) or 30 µM NS11021 (open circles). Lines represent fits with Scheme 2 using parameters in Table 3: red line for 0 µM NS11021; solid blue line for 30 µM NS11021 adjusted for γ δ; dashed blue line for 30 µM NS11021 adjusted for α, β, γ, δ, and *D*. **(D)** τ versus voltage as in C with solid lines showing fits with Scheme 2 using parameters in Table 3: red line for 0 µM NS11021; green line for 30 µM adjusted for α β; purple line for 30 µM adjusted for *D*. Adjusting α and β or *D* can only describe the gating kinetics with 30 µM NS11021 at negative voltages (less than −100 mV) when in combination with adjustment of γ and δ.

Simultaneous (global) fitting was performed using G–V relations over a range of Ca^2+^ with 0 µM NS11021, as described in Materials and methods. G–V relations predicted by above parameters in Fit A and Fit B can be found in Figs. 7 A and 8 A, respectively. χ^2^ values for comparison of experimental data versus corresponding prediction (based on 90 experimental data points): Fit A, 0.045; Fit B, 0.044.

Using this approach, we observed that in the case of both parameter sets (Fit A and Fit B), fits with three of the individual parameters, *L*_0_, *J*_0_, and *D*, provided nearly equivalent descriptions of the experimental G–V-Ca^2+^ relations (Fig. 7 and bold values in Table 2). As an example of how much these parameters were affected by the drug, *L*_0_, *J*_0_, and *D* were altered by 2.6-, 1.5-, and 1.3-fold for Fit A and 2.2-, 1.6-, and 1.5-fold for Fit B per threefold increase in [NS11021] between 10 and 30 µM NS11021 (Fig. 7 F and Figs. S2, S3, S4, and S5). Although these results could be interpreted to mean that effects on *L*_0_, *J*_0_, or *D* were approximately equally likely to underlie NS11021 action in the channel, we had previously reasoned that the NS11021 activation must, at a minimum, affect *L*_0_ (which governs the C-O equilibrium at the PGD) because NS11021 altered the gating at very negative voltages at which the VSD is largely at rest.

**Table 2.** Results of changing individual parameters in Scheme 1 to account for effects of NS11021

Parameter	Fit A		Fit B	
	Value at 30 µM NS11021	χ^2^	Value at 30 µM NS11021	χ^2^
*J*_0_	**0.32**	**1.96**	**0.36**	**2.53**
*K_D_* (µM)	6.8	11.8	2.9	11.29
*L*_0_	**8.7 × 10^−5^**	**2.16**	**2.1 × 10^−4^**	**3.77**
*z_J_* (e_0_)	0.82	11.35	1.3	9.14
*z_L_* (e_0_)	1.8	11.52	2.0	12.09
*C*	6.0	10.40	35	11.66
*D*	**74**	**2.37**	**61**	**1.56**
*E*	5.0	9.46	5.1	9.86

Simultaneous (global) fitting was performed using G–V relations acquired over a range of Ca^2+^ for [NS11021] at 0, 0.1, 1, 10, and 30 µM, with parameters shown above adjusted individually, as described in Materials and methods. Adjusted parameter values at 30 µM NS11021 are shown above, along with summed χ^2^ values for fitting over the entire range of [NS11021] and [Ca^2+^] (based on 410 experimental data points). Unadjusted parameter values (corresponding to 0 µM NS11021) can be found in Table 1. The bold values emphasize the parameters *L_0_*, *J_0_*, and *D* that, when changed, provided nearly equal descriptions of the experimental G-V-Ca^2+^ relations.

To further explore the validity of the three best single-parameter fits with Scheme 1, we first calculated the P_o_ values predicted by Scheme 1 at nominally 0 Ca^2+^ and Vm less than or equal to −60 mV, for 0 and 30 µM NS11021, using the values for *L*_0_, *J*_0_, and *D* estimated as described above. Here we reasoned that the more valid parameter set should yield a better prediction of these P_o_ data with 30 µM NS11021. Among these, Scheme 1 with *L*_0_ adjusted to account for the effects of increasing [NS11021] yielded the best description of these P_o_ data at which the CSD and VSD are predicted to be largely in the resting state (Fig. 8, A and B).

Although consistent with the idea that an increase in *L*_0_ could describe the effects of NS11021 on BK channel gating, the results determined from predictions of Scheme 1 are not unequivocal. Thus, we reasoned that a more stringent test of model validity would be to use a reduced kinetic scheme, Scheme 2, to describe the voltage-dependent activation and deactivation time constants that had been estimated with nominally 0 Ca^2+^, and then identify a set of parameters for Scheme 2 that can account for the effects of NS11021 on the time constants (Marks and Jones, 1992; Horrigan et al., 1999). Scheme 2, in principle, should account for the major features of voltage-dependent BK channel gating in the absence of Ca^2+^, and so it should be possible to identify which specific rate constants in Scheme 2 best account for the effects of NS11021. Using this approach, we first identified a set of parameters (rate constants and their corresponding voltage dependences) to describe the τ versus *V* relation at 0 Ca^2+^, in the absence of NS11021. We then used these parameters as a starting point to find which rate constants might be altered to best describe the τ versus *V* relation with 30 µM NS11021.

Using this approach, we found that it was possible to describe the τ versus *V* relation with 30 µM NS11021 by increasing δ (the rate constant for the C-O transition) by 1.6-fold and decreasing γ (rate constant for C-O transition) by 5.5-fold (Fig. 8 C and Table 3). Because the rates in Scheme 2 are constrained additionally by kinetic data at very negative voltages (to −240 mV, which are not used in determining the parameters of Scheme 1), direct comparison between estimates of the equilibrium constant *L*_0_ from Scheme 1 (2.8 × 10^−6^ for Fit A, 1.0 × 10^−5^ for Fit B) and the ratio of δ/γ (2.7 × 10^−4^) with 0 µM NS11021 reveals discrepancies between the two approaches. However, addition of 30 µM NS11021 resulted in a ninefold increase in the ratio of δ/γ (to 2.4 × 10^−3^) in Scheme 2, which is comparable to the increases in *L*_0_ observed with 30 µM NS11021 using Scheme 1 (30- and 20-fold for Fits A and B, respectively). Whereas these results reveal potential limitations of these simplified gating models, they are consistent with the observation that the major effects of NS11021 can be described by a change in *L*_0_. In contrast, changing only α and β (which underlie the equilibrium constant *J*_0_ from Scheme 1) or changing the allosteric constant *D* (corresponding to *f*^2^ in Scheme 2) could not recapitulate the effects of increasing [NS11021] on time constants at negative voltages (Fig. 8 D).

**Table 3.** Fitted parameters for Scheme 2 constrained by time constants acquired with nominally 0 Ca^2+^, and either 0 or 30 µM NS11021

Parameter	0 µM	30 µM: α β γ δ *D*	30 µM: γ δ	30 µM: α β	30 µM: *D*
α (s^-1^)	1,162	**3,387**	1,162	**4,833**	1,162
β (s^-1^)	31,020	**17,200**	31,020	**11,230**	31,020
γ (s^-1^)	5,128	**1,154**	**925.0**	5,128	5,128
δ (s^-1^)	1.40	**1.45**	**2.23**	1.40	1.40
*z*_α_ (e_0_)	0.31	0.31	0.31	0.31	0.31
*z*_β_ (e_0_)	−0.31	−0.31	−0.31	−0.31	−0.31
*z*_γ_ (e_0_)	−0.025	−0.025	−0.025	−0.025	−0.025
*z*_δ_ (e_0_)	0.29	0.29	0.29	0.29	0.29
*D*	8.2	**5.2**	8.2	8.2	**25.6**
χ^2^	13.4	14.9	82.5	167.4	209.7

Time constants of activation/deactivation over voltages ranging from −240 to 230 mV, with nominally 0 Ca^2+^ and 0 µM NS11021 used to estimate parameters for Scheme 2, as described in Materials and methods. These were used as a base set of parameters to describe the time constants in the presence of 30 µM NS11021, by adjusting parameters corresponding to *J*_0_ (α, β), *L*_0_ (γ, δ), or *D*. These adjusted values are shown in bold. Thus, adjusting γ and δ yielded the lowest χ^2^ value among these three comparisons, although adjusting these parameters in combination yielded a statistically better fit. Time constants predicted by these parameters superimposed on experimental data are shown in Fig. 8. χ^2^ based on 41 experimental data points each for 0 and 30 µM NS11021.

### Potential NS11021 actions at other gating modules

Although the above results suggest that the effects of NS11021 cannot be explained without a direct effect at the PGD, we could not rule out the possibility that NS11021 might also affect VSD activation in combination with an effect at the PGD. To test this idea, we performed additional fitting with Scheme 1 over a range of [NS11021] using the parameters illustrated in Table 1 and values for *L*_0_ in Fig. 7 F, while allowing either *J*_0_ or *D* to be adjusted as a function of [NS11021]. These results (summarized in Table S2) show that with the parameters of Fit A, adjusting either *J*_0_ or *D* did not improve the description of the data beyond the fit determined by adjusting only *L*_0_ (shown in Fig. 7).

However, the adjusting either *J*_0_ or *D* in combination with *L*_0_ did result in a marginally better description of the data using parameters from Fit B. Specifically, slight increases in *J*_0_ from 0.06 to 0.073 and 0.085 at 10 µM and 30 µM NS11021, respectively, yielded better descriptions of the G–V relations at these higher NS11021 concentrations (Fig. S6). Likewise, increasing *D* from 16 to 19 and 21 yielded better descriptions of the G–V relations at the same higher NS11021 concentrations (Fig. S7). These results seemed to suggest that the additional actions of NS11021 may be detectable at these higher drug concentrations. These effects could arise either from the actions of NS11021 at a single site at the interface between the PGD and VSD or from separate actions of NS11021 through at least two different sites, with one at the PGD and a lower-affinity site at the VSD. The possibility of a mechanism involving the VSD is supported by a fit with Scheme 2 that allows α, β, γ, δ, and *D* to all be adjusted to account for the gating kinetics at 30 µM NS11021 (illustrated in Table 3 and Fig. 8 C, dashed blue line). This fit yielded a description of the data that was especially improved at depolarized voltages (where the VSD is activated) compared with the best fit with adjustment of only γ and δ (Fig. 8 C, solid blue line).

To further explore the possibility of NS11021 effects on VSD activation, we performed further fitting of Scheme 2 by allowing γ and δ to be adjusted in combination with either α and β or *D*. Each of these combinations yielded marginally better descriptions of the gating kinetics with 30 µM NS11021 than fitting γ and δ alone (Fig. S8 and Table S3), again supporting the idea that NS11021 may impact VSD activation at higher drug concentrations (>10 µM).

Although the robust activating effects of NS11021 in the nominal absence of Ca^2+^ and on truncated BK channels from which the CSD is deleted suggest that NS11021 actions do not require the CSD, examination of the *V*_1/2_ versus [NS11021] relation suggests that the spacing between G–V curves as a function of [Ca^2+^] may be slightly decreased at high drug concentrations, and this action is not predicted by an increase in *L*_0_ alone with increasing [NS11021] (Fig. S9). This opens that possibility that higher concentrations of NS11021 may also have some impact on Ca^2+^ sensitivity of the channel. To test this idea, we performed additional fitting with Scheme 1 over a range of [NS11021] using the parameters illustrated in Table 1 and values for *L*_0_ in Fig. 7 F, while allowing either *K_D_* or *C* to be adjusted as a function of [NS11021]. These results (summarized in Table S2) show that with either the parameters of Fit A or Fit B, adjusting *C* did yield marginal improvement in the description of the data beyond the fit determined by adjusting only *L*_0_ (Figs. S10 and S11, respectively). The values determined for *C* were not well conserved between these two parameters sets; however, interestingly, the fractional change in *C* as a function of increasing [NS11021] was remarkably similar, with a 16% decrease (from 2.5 to 2.1) for Fit A and 12% decrease (from 17 to 15) for Fit B over the range of 0–30 µM NS11021. Together, these results are consistent with the idea that the most consistent and salient effect of NS11021 is at the PGD, whereas we cannot rule out the possibility of actions that may involve activation of the VSD and slightly decreased coupling of the CSD at higher NS11021 concentrations.

## Discussion

### Mechanism of NS11021 activation

Our results are consistent with the idea that NS11021 facilitates voltage-dependent BK channel opening primarily by slowing the deactivation kinetics of the channel. This is achieved through an action that does not strictly require the presence of the CSD or activation of the VSD, and thus at a minimum involves a direct action at the PGD.

In terms of mechanism, if we assume that a single NS11021 molecule is required to act on the channel to drive the C-O equilibrium toward the open state, then the experimental data suggest that each NS11021 molecule may shift the C-O equilibrium toward the open state by at least ninefold (2.4 × 10^−3^/2.7 × 10^−4^). In this case, the activating mechanism of NS11021 for channels with both the VSD and CSD at rest can be summarized by a four-state model (Scheme 3).

**(Scheme 3):**
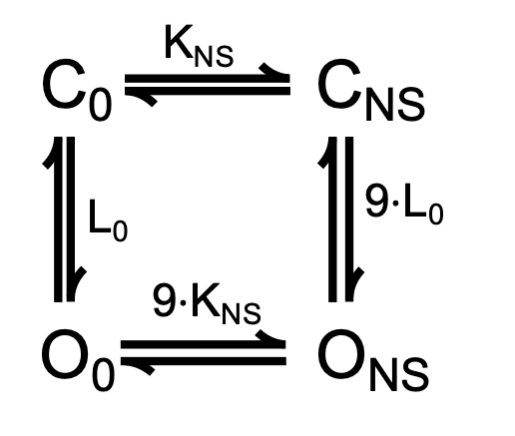


Thus, the NS11021 affinity is approximately ninefold higher in the open state compared with the closed state. Based on this scheme, we can make a minimal estimate of the energetics of NS11021 action using ΔΔ*G_NS_*_11021_ = −*RT*ln(9) = −1.3 kcal/mol. This estimate is based on rate constants determined from kinetic data obtained at 0 and 30 µM NS11021 using Scheme 2. Lower-limit estimates of this NS11021 coupling factor using Scheme 1, constrained by steady-state kinetic data, are 21 and 31 for Fit A and Fit B, respectively (ΔΔ*G_NS_*_11021_ = −1.8 and −2.0 kcal/mol). Together, these estimates can thus provide a range for the energetic impact of NS11021 on gating of the BK channel pore.

Because of the limited aqueous solubility of NS11021, it was not possible to perform reliable experiments with [NS11021] >30 µM, making it difficult to estimate the EC_50_ for the drug. However, based on dose–response relations obtained over a range of voltages and [Ca^2+^], it was possible to estimate EC_50_ values for NS11021 that ranged up to 28 µM, as defined in our analysis (Fig. 6). Thus, if one assumes that BK channels gate primarily between one open and one closed state in the absence of NS11021, then Scheme 3 would predict that the channel would gate among at least two open and two closed states with 30 µM NS11021. Consistent with this prediction, we observed an increase in the numbers of open and closed states in these conditions (Fig. 5), which may thus reflect gating among NS11021-bound and unliganded open and closed conformations.

### Physical mechanism of NS11021 activation

Permeant ions such as Rb^+^ are observed to increase the P_o_ of K^+^ channels. Rb^+^ can enter the K^+^ channel selectivity filter and is thought to stabilize the open state because of its higher affinity for the pore relative to K^+^ (Swenson and Armstrong, 1981; Demo and Yellen, 1992). This mechanism of activation for Rb^+^ and other permeant ions has been described as a “foot in the door” (Mienville and Clay, 1996; Swenson and Armstrong, 1981; Thompson and Begenisich, 2012; Piskorowski and Aldrich, 2006; Demo and Yellen, 1992). Similar to NS11021, Rb^+^ activates BK channels and is observed to slow the closing rate while not substantially impacting the opening rate to increase the single-channel mean open time. Recently it was proposed that several K^+^ channels (including BK, TREK-1, and hERG) are activated by negatively charged activators (NCAs) such as NS11021 and permeant ions such as Rb^+^ through a related mechanism (Schewe et al., 2019). Specifically, it was hypothesized that NS11021 binds in a region lining the cavity of the channel, where the negatively charged tetrazole moiety of NS11021 can interact with permeant ions, and can thus promote K^+^ binding within the pore cavity and stabilize the open state. This model would suggest that NS11021 might increase the single-channel conductance in BK channels, as observed with the action of the NCA BL-1249 on TREK-2 channels (Schewe et al., 2019). In our experiments, we did not observe a substantial increase in BK single-channel conductance at negative voltages, whereas the mean open times were increased (Fig. 4 C). Thus, the physical mechanism for NS11021 action in BK channels may be less clear than the mechanism of NCAs in TREK-2 channels, and resolving the structural basis for NCA action in BK channels may require more direct structural measurements.

### Limitations of the proposed mechanism

Although increasing *L*_0_ with increasing [NS11021] in the context of Scheme 1 can describe the major features of NS11021 action on BK channel gating in our experiments, we observe that the model falls short of the best possible description of the data at higher [NS11021]. Specifically, Scheme 1 predicts slightly greater Ca^2+^-dependent shifts in *V*_1/2_ values at 10 µM and 30 µM NS11021 than are observed in the experimental data (Fig. 7, D and E; and Fig. S9). We have presented model fits using two different sets of parameters, and we have minimized the possibility that we have not identified the best parameters by refitting Scheme 1 many times with different sets of starting parameters. Nonetheless, it is possible that there are additional, unknown sets of parameters that might yield better descriptions of the data.

The observation that changing only *L*_0_ is insufficient to perfectly recapitulate the Ca^2+^-dependent spacing among G–V relations at higher [NS11021] further leaves open the possibility that at higher drug concentrations, NS11021 may act at alternative sites to effect voltage or Ca^2+^ sensing. The presumed lower-affinity NS11021 sites may be poorly accessible from the aqueous solution, or they may be characterized by weak chemical interactions to yield a rapid off-rate. It is also possible that the slightly decreasing effect of high [NS11021] on *V*_1/2_ values with high [Ca^2+^] (illustrated in Fig. S9) could be that activation of the Ca^2+^-sensor allosterically inhibits the action of NS11021, through an action distinct from the activating effect of the drug.

While direct assessment of allosteric coupling between the VSD and CSD (parameter *E*) has historically been challenging, it was recently hypothesized that VSD–CSD coupling may have a substantial impact on voltage-dependent activation (Lorenzo-Ceballos et al., 2019; Hite et al., 2017). Consistent with this idea, although both Fit A and Fit B describe G–V-Ca^2+^ relations similarly well at 0 µM NS11021 (Table 1), changing *L*_0_ in Fit A better accounts for the observed effects of NS11021 at higher drug concentrations and contains an 18-fold greater value for parameter *E* than Fit B (Fig. 8 A, Fig. S6 A, and Table 1).

It is important to acknowledge that Scheme 1 itself is a simplification of the underlying gating mechanism, as it is well established that BK channels contain multiple Ca^2+^ binding sites on each channel subunit that are not accounted for or described by Scheme 1. However, fitting with a more complex model with multiple Ca^2+^ binding sites would give rise to additional difficulty in identification of parameters, as the additional parameters would be poorly constrained by our experimental data. Scheme 1 should thus be considered a working hypothesis. Similarly Scheme 2 should be considered a simplification of gating in the absence of Ca^2+^, as it cannot account for gating transitions of the CSD that could arise from infrequent Ca^2+^ binding events at very low [Ca^2+^]. In addition, simplifying assumptions are made in the parameterization of both Schemes 1 and 2; for example, voltage- and Ca^2+^-dependent transitions in these schemes are modeled as being independent for each subunit, whereas they may exhibit cooperativity (Niu and Magleby, 2002; Qian et al., 2006; Shelley et al., 2010). Despite these shortcomings, Schemes 1 and 2 enable the quantitative testing of mechanisms that support the idea that NS1102 acts relatively selectively at C-O gating transitions.

### Conclusions

In the present study, we show that NS11021 activation of BK channel is largely due to stabilization of an open state at the PGD. Whereas it has been hypothesized that NS11021 binds within the cavity of the channel (Schewe et al., 2019), the actions of the small-molecule activators Cym04 and NS1619 appear to depend on the integrity of the C-linker of the BK channel and can be disrupted by single amino acid substitutions in that region (Gessner et al., 2012). The functional effects of Cym04 and NS1619 also appear distinct from those of NS11021: Cym04 and NS1619 primarily act by shifting the voltage-sensor equilibrium toward the activated state (increasing *J*_0_ in Scheme 1), whereas the P_o_ is altered little under conditions where the VSD is at rest. It will be important to identify molecular mechanisms of additional structural classes of BK channel targeting drugs to improve our understanding of BK channel gating, as well as the development of novel therapies to treat disease.

## Supplementary Material

Table S1 shows mean values of *V*_1/2_ and *z* determined from Boltzmann fits of individual G–V relations using Eq. 1.

Table S2 shows the results of changing *L*_0_ plus a second parameter in Scheme 1 to account for effects of NS11021.

Table S3 shows additional sets of fitted parameters for Scheme 2 constrained by time constants acquired with nominally 0 Ca^2+^ and either 0 or 30 μM NS11021.
