# Supplementary material for: Molecular mechanism of BK channel activation by the smooth muscle relaxant NS11021
Source: J Gen Physiol. 2020 Mar 27;152(6):e201912506. doi: 10.1085/jgp.201912506 (PMC7266150; doi:10.1085/jgp.201912506)
Supplement: Table S1 — shows mean values of V1/2 and z determined from Boltzmann fits of individual G–V relations using Eq. 1. [file JGP_201912506_TableS1.docx]

| **Supplemental Table 1.** Mean values of V_1/2_ and z determined from Boltzmann fits of individual G-V relations using Eq. 1 | | | | |
| --- | --- | --- | --- | --- |
| [NS11021] (μM) | 0 Ca^2+^  V_1/2_ (in mV), (n) | 1 μM Ca^2+^  V_1/2_ (in mV), (n) | 10 μM Ca^2+^  V_1/2_ (in mV), (n) | 100 μM Ca^2+^  V_1/2_ (in mV), (n) |
| 0 | 158.2 ± 1.7 (54) | 110.3 ± 2.1 (26) | 23.4 ± 2.0 (20) | -41.4 ± 4.0 (15) |
| 0.1 | 145.2 ± 5.7 (12) | 105.9 ± 3.2 (14) | 23.1 ± 4.6 (7) | -55.2 ± 5.0 (10) |
| 1 | 129.5 ± 3.7 (4) | 91.1 ± 6.0 (11) | 7.8 ± 2.3 (7) | -72.3 ± 5.0 (7) |
| 10 | 88.5 ± 3.0 (4) | 67.8 ± 4.4 (9) | -8.2 ± 2.3 (6) | -84.0 ± 4.0 (4) |
| 30 | 73.1 ± 5.9 (14) | 43.4 ±3.6 (7) | -38.5 ± 3.4 (6) | -89.4 ± 5.7 (10) |
| [NS11021] (μM) | 0 Ca^2+^  z (e_0_) | 1 μM Ca^2+^  z (e_0_) | 10 μM Ca^2+^  z (e_0_) | 100 μM Ca^2+^  z (e_0_) |
| 0 | 1.39 ± 0.03 | 1.97 ± 0.05 | 1.29 ± 0.05 | 1.27 ± 0.09 |
| 0.1 | 1.53 ± 0.09 | 2.02 ± 0.12 | 1.13 ± 0.10 | 1.02 ± 0.11 |
| 1 | 1.72 ± 0.11 | 1.80 ± 0.15 | 1.22 ± 0.07 | 1.09 ± 0.20 |
| 10 | 1.53 ± 0.30 | 1.53 ± 0.08 | 1.22 ± 0.05 | 1.01 ± 0.20 |
| 30 | 1.30 ± 0.35 | 1.49 ± 0.03 | 1.00 ± 0.05 | 1.21 ± 0.23 |
