# Supplementary material for: Molecular mechanism of BK channel activation by the smooth muscle relaxant NS11021
Source: J Gen Physiol. 2020 Mar 27;152(6):e201912506. doi: 10.1085/jgp.201912506 (PMC7266150; doi:10.1085/jgp.201912506)
Supplement: Table S2 — shows the results of changing L0 plus a second parameter in Scheme 1 to account for effects of NS11021. [file JGP_201912506_TableS2.docx]

| **Supplemental Table 2.** Results of changing L_0_ plus a second parameter in Scheme 1 to account for effects of NS11021 | | | | |
| --- | --- | --- | --- | --- |
| Parameter | Fit A | | Fit B | |
|  | Value at 30 μM NS11021 | 𝜒^2^ | Value at 30 μM NS11021 | 𝜒^2^ |
| J_0_ | 0.08 | 2.16 | 0.09 | 3.21 |
| C | 2.1 | 1.83 | 15 | 3.20 |
| D | 29 | 2.16 | 21 | 3.02 |
| Simultaneous (global) fitting was performed using G-V relations acquired over a range of Ca^2+^ with [NS11021] at 0.1, 1, 10, and 30 μM, with parameters shown above adjusted in addition to L_0_, as described in Methods. Adjusted parameter values at 30 µM NS11021 are shown above, along with summed 𝜒^2^ values for fitting over the entire range of [NS11021] and [Ca^2+^] (based on 410 experimental data points). The other parameters for Scheme 1 at 30 μM NS11021 can be found in Table 1, except L_0_ = 8.7 x 10^-5^ for Fit A and 2.1 x 10^-4^ for Fit B. Changing L_0_ alone resulted in a 𝜒^2^ value of 2.16 for Fit A and 3.77 for Fit B. Starting values for each parameter can be found in Table 1, Figure 7, and Figure 8. | | | | |
