# Supplementary material for: Molecular mechanism of BK channel activation by the smooth muscle relaxant NS11021
Source: J Gen Physiol. 2020 Mar 27;152(6):e201912506. doi: 10.1085/jgp.201912506 (PMC7266150; doi:10.1085/jgp.201912506)
Supplement: Table S3 — shows additional sets of fitted parameters for Scheme 2 constrained by time constants acquired with nominally 0 Ca2+ and either 0 or 30 μM NS11021. [file JGP_201912506_TableS3.docx]

| **Supplemental Table 3.** Additional sets of fitted parameters for Scheme 2 constrained by time constants acquired with nominally 0 Ca^2+^, and either 0 or 30 μM NS11021 | | | | | |
| --- | --- | --- | --- | --- | --- |
| Parameter | 0 µM: | 30 μM:  α β γ δ D | 30 μM:  α β γ δ | 30 μM:  γ δ D | 30 μM:  γ δ |
| α (s^-1^) | 1,162 | **3,387** | **3,202** | 1,162 | 1,162 |
| β (s^-1^) | 31,020 | **17,200** | **30,860** | 31,020 | 31,020 |
| γ (s^-1^) | 5,128 | **1,154** | **1,444** | **854.6** | **925.0** |
| δ (s^-1^) | 1.40 | **1.45** | **1.36** | **2.41** | **2.23** |
| z_α_ (e_0_) | 0.31 | 0.31 | 0.31 | 0.31 | 0.31 |
| z_β_ (e_0_) | -0.31 | -0.31 | -0.31 | -0.31 | -0.31 |
| z_γ_ (e_0_) | -0.025 | -0.025 | -0.025 | -0.025 | -0.025 |
| z_δ_ (e_0_) | 0.29 | 0.29 | 0.29 | 0.29 | 0.29 |
| D | 8.2 | **5.2** | 8.2 | **5.6** | 8.2 |
| 𝜒^2^ | 13.4 | 14.9 | 47.6 | 72.3 | 82.5 |
| Time constants of activation/deactivation over voltages ranging from -240 to 230 mV, with nominally 0 Ca^2+^ and 0 μM NS11021 were used to estimate parameters for Scheme 2, as described in Methods. These were used as a base set of parameters to describe the time constants in the presence of 30 μM NS11021, by adjusting parameters corresponding to J_0_ (α, β), L_0_ (γ, δ), or D (where D = f^2^). These adjusted values are shown in **bold**. Adjusting model parameters in addition to γ and δ yielded lower 𝜒^2^ values, suggesting that VSD activation, or coupling between VSD activation and PGD opening, may be affected by NS11021. Time constants predicted by these parameters superimposed on experimental data are shown in Supplemental Figure 11. 𝜒^2^ based on 41 experimental data points each for 0 and 30 µM NS11021. | | | | | |
